# Structured Scintillators for Efficient Radiation Detection

**DOI:** 10.1002/advs.202102439

**Published:** 2021-11-10

**Authors:** Ziyu Lin, Shichao Lv, Zhongmin Yang, Jianrong Qiu, Shifeng Zhou

**Affiliations:** ^1^ State Key Laboratory of Luminescent Materials and Devices School of Materials Science and Engineering South China University of Technology Guangdong Provincial Key Laboratory of Fiber Laser Materials and Applied Techniques Guangdong Engineering Technology Research and Development Center of Special Optical Fiber Materials and Devices Guangzhou 510640 China; ^2^ College of Optical Science and Engineering Zhejiang University Hangzhou 310027 China

**Keywords:** arrays, fibers, particles, radiation detection, structured scintillators

## Abstract

Scintillators, which can convert high‐energy ionizing radiation into visible light, have been serving as the core component in radiation detectors for more than a century of history. To address the increasing application demands along with the concern on nuclear security, various strategies have been proposed to develop a next‐generation scintillator with a high performance in past decades, among which the novel approach via structure control has received great interest recently due to its high feasibility and efficiency. Herein, the concept of “structure engineering” is proposed for the exploration of this type of scintillators. Via internal or external structure design with size ranging from micro size to macro size, this promising strategy cannot only improve scintillator performance, typically radiation stopping power and light yield, but also extend its functionality for specific applications such as radiation imaging and therapy, opening up a new range of material candidates. The research and development of various types of structured scintillators are reviewed. The current state‐of‐the‐art progresses on structure design, fabrication techniques, and the corresponding applications are discussed. Furthermore, an outlook focusing on the current challenges and future development is proposed.

## Graphical abstract

A comprehensive overview on developing novel scintillators by structure engineering strategy, which focuses on rationally tailoring the structures with typical size spanning micro, meso, and macroranges, is provided. The state‐of‐the‐art progresses on structure design, fabrication techniques, and the corresponding applications are systematically discussed. Future perspectives focusing on current challenges are presented as well.

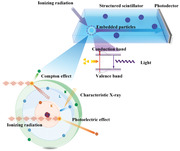

## 1 Introduction

Radiation science and technology play irreplaceable roles in tremendous fields such as nuclear medicine, imaging, high‐energy physics, and homeland security. For instance, a carefully controlled ionizing radiation beam could be tightly focused on a tumor to kill malignant cells in cancer therapy. Monitoring high‐energy cosmic ray provides the majority of universe process information such as dark‐matter particle annihilation and supernova explosions. In the food industry, radiation exposure at a certain dose enables product shelf‐life extension via sterilization and selectively destroying organisms responsible for spoilage and inhibiting sprouting.^[^ ^1^ ^]^ For all these applications, a fundamental prerequisite is the accurate detection of incident radiation. To meet the basic requests of practical application, various technologies have been developed in the past few decades, among which the indirect conversion approach based on scintillators is one of the most popular methods.

Scintillators refer to a kind of material that can convert invisible high‐energy photons (e.g., X‐ray or gamma‐ray) or particles (e.g., electrons, protons, neutrons, or heavy ions) to a flash of light. The overall scintillation can be mainly divided into three stages: conversion, transport, and emission. In the conversion stage, incident radiation interacts with the scintillator and deposits their energy through photoelectric effect, Compton scattering, and pair production. Specifically, uncharged particles such as neutrons must first convert their energy into secondary charged particles, and then they can ionize the scintillator. For X‐ray or gamma ray, radiation–material interaction probability, namely, radiation stopping power, increases with scintillator density and effective atomic number (*Z*). While for uncharged neutrons, neutron–material interaction probability, normally known as neutron cross‐section, is element and isotope (^3^He, ^6^Li, ^10^B, ^133^Cd, ^155^Gd, and ^157^Gd) dependent. As a consequence of the conversion stage, hot electrons and deep holes are formed and gradually thermalized. Deposited energy can then be transferred to luminescent centers by the migration of electron–hole pairs and excite the luminescent centers. Finally, excited luminescent centers return to the ground state and emit the desired scintillation light. By coupling the scintillators to a photodetector, the emitted light can be collected and converted into electronic signal for further analysis.^[^ ^2^ ^]^

According to the described mechanism, scintillators govern the performance of the radiation detecting system. Since the invention of the first scintillator, CaWO_4_, which was used for the discovery of X‐ray in 1895, the family members of scintillators rapidly increased. Numerous scintillators emerged, and mature products with outstanding performances are already on the market. The developments of scintillators have been reviewed in several outstanding papers and books, including but not limited to those by Nikl and co‐workers,^[^ ^3^ ^]^ Lecoq and co‐workers,^[^ ^4^ ^]^ Dujardin and co‐workers,^[^ ^5^ ^]^ Pei and co‐workers,^[^ ^6^ ^]^ and McGregor and co‐workers.^[^ ^7^ ^]^ The criteria of evaluating a scintillator are normally based on the following parameters: scintillation efficiency, light yield, radiation stopping power, decay times, energy resolution, and radiation harness. In practical applications, several other parameters, including cost, large‐volume production possibility, chemical stability, mechanical strength, and scintillator–photodetector coupling efficiency, must be considered as well. Driven by the rapid development of radiation science and technology, the research on seeking next‐generation scintillators with better performance remains an active topic and a difficult challenge.

**Figure 1:**
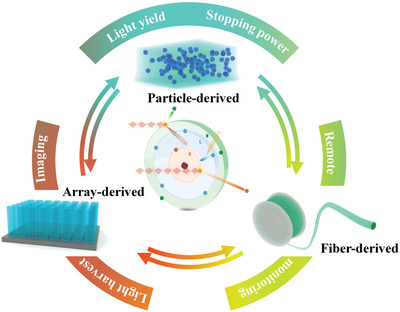
Structure engineering toward next‐generation scintillators.

The classic strategies for developing scintillators mainly lie on discovering novel material candidates and improving existing traditional scintillators, which are homogeneous in chemical composition and structure without any secondary phase. They generally appear in the form of bulk shape and size. The most popular way to explore and improve the scintillators involves the “composition engineering” strategy, which targets material electronic band manipulation, and the “defect engineering” strategy, which aims to tailor the effect of beneficial or deleterious defects.^[^ ^8^ ^]^ Given that the performance of a scintillator is remarkably affected by its microstructure, “structure engineering,” which focuses on rationally tailoring the structures with typical size spanning micro‐, meso‐, and macroranges, has been demonstrated as another efficient, feasible strategy to develop high‐performance scintillators and arouse increasing attention(Figure 1). Via microstructure engineering such as second‐phase particle introduction, structured scintillators with a strong performance can be constructed and even functionalized with specific performance that can hardly be obtained by traditional scintillators. Through meso‐ and macrostructure engineering, special structured scintillators with a large radiation sensing area composed of small units can be constructed, and they enable high‐resolution imaging and remote radiation monitoring. Despite the importance of the “structure engineering” strategy in developing new scintillators, to the best of our knowledge, no review paper specifically focuses on such a promising strategy.

In this review, the advances of the application of the “structure engineering” strategy for construction of new generated structured scintillators are highlighted. According to their characteristics, structured scintillators are divided into three groups and introduced in the following paragraphs: particle‐, array‐, and fiber‐derived structured scintillators. Following the brief introduction (Section 1), particle‐derived structured scintillators obtained by embedding various particles in polymer or glass are introduced (Section 2). Then, the progress in array‐derived structured scintillators constructed via rational generation of array inside or on the surface of the scintillators is presented (Section 3). Afterward, fiber‐derived structured scintillators built via thermal drawing are summarized and discussed (Section 4). In the final section (Section 5), alongside a brief conclusion of this review, an outlook on the future development is presented.

## 2 Particle‐Derived Structured Scintillators

Inorganic single crystals are probably the most widely used scintillators in practical applications over the past decades. For instance, the majority of scintillators serving in CT scanners by far is still CdWO_4_ single crystals, and NaI:Tl crystals dominate the measurement of gamma rays in oil well logging.^[^ ^9^ ^]^ Nevertheless, the difficulty in producing large volume sample and cost consideration seriously compromise the large‐area application of crystal scintillators, especially for those with hygroscopicity. Other alternatives are needed. Glass and plastic scintillators can be promising candidates for their advantages of low cost, large volume production possibility, and controllable shape, but they suffer from relatively low light yields.^[^ ^10^ ^]^ Immense efforts have been devoted to improving scintillating performance. Other than directly modifying the component elements, introducing particles into matrix as a second phase to construct particle‐derived structured scintillators was more recently employed as a low‐cost but effective strategy.

### 2.1 Particle–Polymer Structured Scintillators

Generally, plastic scintillators consist of polymer matrix such as commercially available polystyrene (PS) and polyvinyl toluene (PVT), and fluorescent dye whose energy levels match the matrix for achievement of efficient energy transfer.^[^ ^11^ ^]^ When a high‐energy particle interacts with plastic scintillators, energy could deposit in the polymer matrix and ionize the dye to emit fluorescence. For common X‐ray or gamma‐ray detection, radiation stopping ability is mainly dependent on density and *Z*. However, polymer exhibits intrinsic low density and *Z* compared with inorganic materials. Owing to the difficulty in the direct synthesis of the polymer matrix composed of high heavy elements, loading high *Z* particles such as inorganic crystals and quantum dots as a second phase into the polymer matrix is a promising alternative strategy.

To incorporate inorganic particles into the polymer matrix, the basic requirements of optical transparency must be first fulfilled. Neglecting the effect of material absorption, Rayleigh scattering caused by embedded particles is the dominating cause of transmission loss, which can be described as follows:^[^ ^12^ ^]^

(1) $$\frac{I}{I_{0}}=e^{-\left[\frac{3\Phi_{p}xr^{3}}{4\lambda^{4}}\left(\frac{n_{p}}{n_{m}}-1\right)\right]}\;$$

where *I* is the intensity of transmitted light, *I*_0_ is the intensity of incident light, *r* is the radius of embedded particles, *n*_p_ is the refractive index of embedded particles, *n*_m_ is the refractive index of polymer matrix, *Φ*_p_ is the volume fraction of particles, and *x* is the optical path length. According to Equation 1, two general strategies can be adopted to design the transparent composites: 1) minimizing particle size *r* and 2) matching refractive indexes of matrix and particles to attain *n*_p_ = *n*_m_. Given that refractive index is intrinsically related to density and electronic polarizability, polymer matrix exhibits certain values of refractive index, making refractive index matching difficult. In this case, controlling particle size seems preferable. Normally, to eliminate the effect of Rayleigh scattering, particle sizes in nanoscale are required. However, embedding nanoparticles into polymer matrix without aggregation is highly challenging due to the high specific surface area and surface energy of nanomaterials. Strategies such as surface modification pretreatment and in situ polymerization are preferred to be adopted, rather than direct mixing.

**Figure 2:**
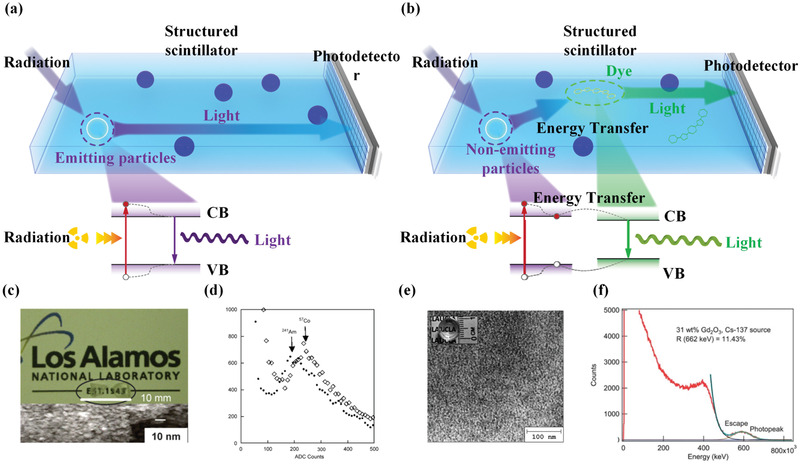
Schematic diagram of particle–polymer structured scintillators incorporated with a) emitting particles and b) nonemitting particles. c,d) Photograph and pulse height spectrum of structured scintillator incorporated with emitting LaF_3_:Ce particles. e,f) TEM image and pulse height spectrum of structured scintillator incorporated with nonemitting Gd_2_O_3_ particles and dye. Inset: photograph. c,d) Reproduced with permission.^[^ ^28^ ^]^ Copyright 2007, Elsevier. e,f) Reproduced with permission.^[^ ^19^ ^]^ Copyright 2013, Royal Society of Chemistry.

Guided by the above principle, various types of inorganic crystals, including halide families such as BaF_2_,^[^ ^13^, ^14^ ^]^ CeF_3_,^[^ ^15^, ^16^, ^17^ ^]^ and CsI;^[^ ^13^, ^18^ ^]^ oxide family such as Gd_2_O_3_,^[^ ^19^, ^20^, ^21^ ^]^ Lu_2_SiO_5_ (LSO),^[^ ^22^ ^]^ and LaPO_4_;^[^ ^23^ ^]^ and quantum dots such as ZnS,^[^ ^24^ ^]^ ZnO,^[^ ^25^ ^]^ CdSe,^[^ ^26^ ^]^ and CdTe,^[^ ^27^ ^]^ have been selected to be integrated into polymer matrix to construct particle–polymer structured scintillators. Depending on their functions in composites, the incorporated particles can be mainly divided into two groups: emitting particles (**Figure** **2**a) and nonemitting particles (Figure 2b).

**Figure 3:**
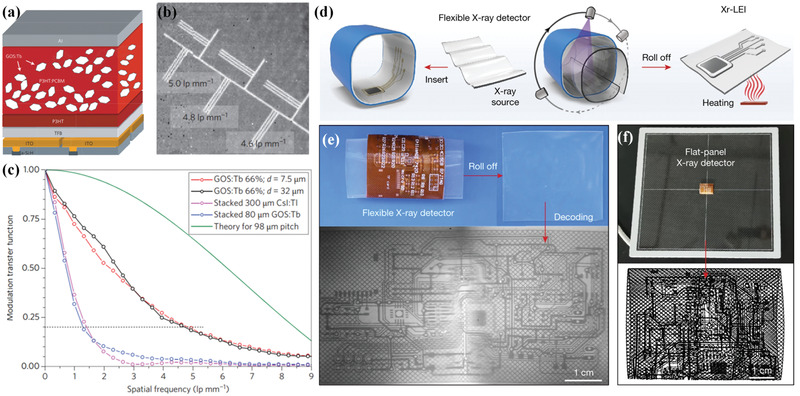
a–c) X‐ray image sensor based on structured scintillator embedded with GOS particles. a) Schematic of image sensor with an a‐Si:H backplane and a hybrid frontplane. b) 70 kV X‐ray image (magnified to region of interest) of a resolution test target. c) MTF of images with different layer thicknesses and two conventional indirect X‐ray converters. Theoretical limit is determined by pixel size. d–f) Flexible X‐ray detector based on structured scintillator embedded with lanthanide‐doped nanoparticles. d) Schematic showing imaging of curved 3D objects. e) Imaging of a 3D electronic board using a prototype detector and f) conventional flat‐panel X‐ray detector. a–c) Reproduced with permission.^[^ ^29^ ^]^ Copyright 2015, Springer Nature. d–f) Reproduced with permission.^[^ ^30^ ^]^ Copyright 2021, Springer Nature.

In particle–polymer structured scintillators with emitting particles, the entire scintillation is completed inside the embedded particles while polymer matrix mainly provides a stable environment and serves as a binder. In early work, typical emitting particles such as CsI, NaI, LaF_3_, Gd_2_O_3_, and Y_2_SiO_5_ were investigated. For example, micrometer‐size emitting particles, including Bi_4_Ge_3_O_12_ (BGO), CsI:Na, Gd_2_SiO_5_:Ce (GSO), CeF_3_, and BaF_2_, were synthesized by simple mechanical grinding of the corresponding single crystals and incorporated into organic matrix, BC‐600.^[^ ^13^ ^]^ Comparison of scintillation decay of the synthesized structured scintillator with the single crystals and emitting particles was discussed in this work. Owing to the large particle size and mismatch of refractive index, all structured scintillators suffered from low transparency, and light yield can hardly be measured. Considering the effect of light scattering induced by embedded particles, nanoscale LaF_3_:Ce emitting particles were incorporated in an organic matrix by McKigney et al.^[^ ^28^ ^]^ In Figure 2c), transmission electron microscopy (TEM) indicates that the size of emitting particles is smaller than 10 nm, ensuring the high transparency of the obtained structured scintillator. Under radiation from ^241^Am and ^57^Co, the as‐made samples displayed the corresponding photopeaks (Figure 2d) but with poor light yields and a low energy resolution. In 2014, a Gd_2_O_3_/poly(vinylidene fluoride, PVDF) structured scintillator was fabricated by Martins and Mendez for X‐ray detection.^[^ ^20^ ^]^ In addition to the improvement of density and *Z*, the introduction of Gd_2_O_3_:Eu nanoparticles in PVDF matrix increased Young's modulus and dielectric constants, facilitating a high carrier mobility and a low nonradiative recombination probability, and resulting in a high energy resolution. Toward medical X‐ray imaging, a quasi‐direct X‐ray detector prototype based on structured scintillator was fabricated via spray coating of Gd_2_O_2_S:Tb (GOS)/organic matrix suspension on the interlayer of a bulk heterojunction (**Figure** **3**a).^[^ ^29^ ^]^ The effect of later spreading of scintillating light could be minimized because the emitted photons of a scintillation can be absorbed by the bulk heterojunction within hundreds of nanometers, much below the pixel size of backplane matrix. This process enabled a spatial resolution of 4.75 lp mm^‐1^ at modulation transfer function (MTF) of 20%. Furthermore, the solution‐based feature of fabrication showed the possibility of producing a large area X‐ray detector with a low cost. Recently, by embedding lanthanide‐doped nanoparticles into polydimethylsiloxane matrix, a flexible structured scintillator was demonstrated by Liu and co‐workers.^[^ ^30^ ^]^ Radiation‐triggered anionic migration in host lattices resulted in the slow hopping of trapped electrons and consequently, a persistent radioluminescence. Figure 3d–f shows that combining with graphical simulation technology, monitoring the afterglow luminescence after X‐ray irradiation enables high‐resolution imaging of curved 3D objects. The proposed structured scintillators show great potential in developing wearable radiation detectors.

Particle–polymer structured scintillators with luminescent quantum dots have also been studied due to their ultrahigh quantum efficiency and luminescent wavelength tunability. For example, Kang et al. demonstrated an X‐ray scintillating screen made of CdTe/polyvinyl alcohol structured scintillator.^[^ ^15^ ^]^ Unique features of CdTe quantum dots enable a bright X‐ray‐induced luminescence and a high‐resolution X‐ray imaging. A spatial resolution of 5 lp mm^‐1^ could be obtained in the imaging experiment, nearly twice than that of a commercial GOS screen (2.8 lp mm^‐1^). In another work on ZnO:Ga/PS structured scintillator reported by Cuba et al., a bright, ultrafast luminescence, below 18 ps, was demonstrated.^[^ ^25^ ^]^ Benefiting from the nonradiative energy transfer from PS matrix to ZnO:Ga quantum dots, the luminescence of PS matrix was quenched, and at least 90% of the energy deposited in PS matrix could be transferred to ZnO:Ga quantum dots in picosecond scale and finally converted into ultrafast luminescence.

**Figure 4:**
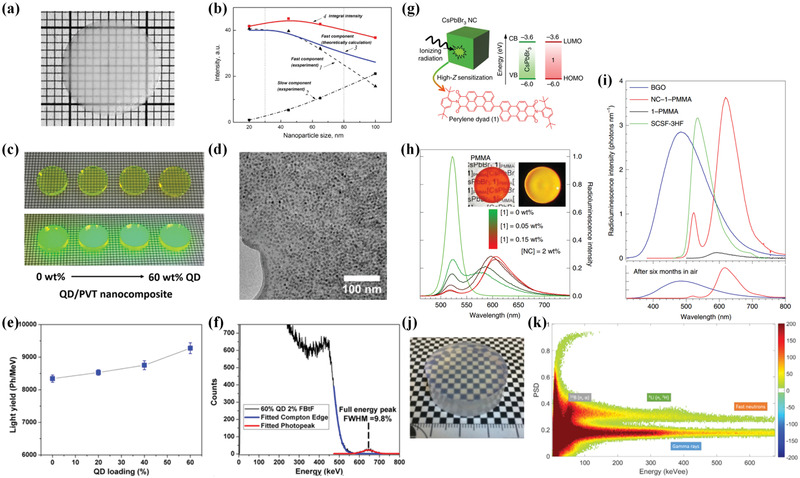
a) Photograph of structured scintillator incorporated with nonemitting BaF_2_ nanoparticles and b) dependence of luminescence intensities on X‐ray excitation of different particle sizes. c–f) Bulk transparent structured scintillators embedded with Cd*_x_* Zn_1‐_ *_x_* S/ZnS quantum dots. c) Photograph. d)TEM image. e) Chart of scintillation light yields. f) Pulse height spectrum. g–i) Structured scintillator incorporated with CsPbBr_3_ quantum dots. g) Energy level of embedded CsPbBr_3_ quantum dots and dye. h) Photoluminescence spectra. Inset: photographs illuminated by ambient light (left) and 400 nm light (right). i) Radioluminescence spectra under X‐ray irradiation (30 kV, 20 mA). j) Photograph of structured scintillator embedded with isotopically ^6^Li_2_^10^B_4_O_7_ nanoparticles and k) subtracted spectra of pulse shape discrimination. a,b) Reproduced with permission.^[^ ^14^ ^]^ Copyright 2016, Elsevier. c–f) Reproduced with permission.^[^ ^34^ ^]^ Copyright 2017, American Chemical Society. g–i) Reproduced with permission.^[^ ^35^ ^]^ Copyright 2020, Springer Nature. j,k) Reproduced with permission.^[^ ^39^ ^]^ Copyright 2019, Royal Society of Chemistry.

The major limitation of above discussed material candidates is that the energy deposited in polymer matrix can hardly be fully utilized in most cases, resulting in a relative low light yield. Moreover, a part of the electrons created by radiation may escape from incorporated particles to polymer matrix and play no contribution on light emission. From this viewpoint, the particle–polymer structured scintillators composed of nonemitting particles and bright luminescent dye are expected to achieve high light yield because of the tunable energy transfer. In these structured scintillators, the ratio of particle average sizes to the mean free path or the thermalization length of electrons is a critical factor that dominates sensitization efficiency. After incident radiation interacts with the material, photoelectrons are formed and gradually thermalized within a certain length. When the mean free path or the thermalization length of electrons exceeds the average size of particle, the majority of photoelectrons could escape from nanoparticles to excite the polymer matrix and the dye, which is known as “electron escape mechanism.” Guided by this principle, a small particle size below thermalization length of electrons is preferred in the presence of energy transfer from embedded particles to dye. To prove this mechanism, Demkiv and co‐workers incorporated BaF_2_ or SrF_2_ nanoparticles with various sizes into PS matrix to demonstrate the effect of particle size.^[^ ^14^, ^31^ ^]^ In **Figure** **4**b, a change of the particle size leads to the redistribution of the fast component (luminescence of dye) and slow component (luminescence of self‐trapped exciton of nanocrystals). Upon increasing the particle size, the fast component decreases while the slow component increases, confirming the existence of energy transfer from particles to matrix through escaped electrons. In a follow‐up work in 2018, a CeF_3_/PS structured scintillator was synthesized with a loading proportion of 40 wt%.^[^ ^17^ ^]^ Rational control of the size of the incorporated particles at 12 nm can help maximize the energy transfer efficiency, resulting in a 16‐fold enhancement of radioluminescence compared with that of pure PS. Particle–polymer structured scintillator via incorporation of nonemitting particles with high neutron cross‐section in polymer matrix was also developed for neutron detection. In this case, nonemitting particles could convert incident neutrons into secondary charged particles and finally transfer deposited energy to the dye. Additionally, intrinsic low *Z* features of plastic scintillator help reduce gamma sensitivity because neutron sources always come up with a gamma background, benefiting avoiding radiation misidentification. For instance, submicron size ^6^LiF crystals were synthesized and distributed into polyvinyl naphthalene with fast decay ADS156FS dye (3 ns) by Sen and co‐workers.^[^ ^32^ ^]^ Considerable light output compared with commercial GS20 neutron detection glass could be obtained. Furthermore, gamma and neutron discrimination can be achieved through pulse height analysis.

Similarly, particle–polymer structured scintillator composed of nonemitting quantum dots and bright luminescent dye has been studied. In this type of material candidates, the existence of energy transfer could not only facilitate the utilization of deposited energy in polymer matrix but also suppress the unexpected self‐absorption of quantum dots. Campbell and Crone first demonstrated Förster excitation transfer from the nonemitting quantum dots to the polymer matrix in 2006.^[^ ^33^ ^]^ CdSe–ZnSe core–shell quantum dots were embedded into a soluble, conjugated polymer poly[2‐methoxy‐5‐(2’‐ethylhexyloxy)‐p‐phenylene vinylene] via spin‐cast mixture solutions in chloroform. Homogeneous samples with various volume fractions of quantum dots (0, 7%, and 15%) were synthesized, and the sample with 15% loading exhibited the brightest cathodoluminescence under electron‐beam excitation, which is more than one order magnitude higher compared with that of undoped polymer. To increase the high energy radiation attenuation further, Pei and co‐workers synthesized a bulk transparent structured scintillator highly loaded with nonemitting quantum dots and FBtF dye, and tested it for gamma‐ray detection.^[^ ^34^ ^]^ Via in situ copolymerization of the partially methacrylate‐functionalized quantum dots in a monomer solution, up to 60 wt% of Cd*_x_* Zn_1‐_ *_x_* S/ZnS core–shell quantum dots can be loaded in PVT matrix with a thickness of 2 mm without inducing substantial transmission loss (Figure 4c–f). Aggregation of quantum dots can be prevented by utilizing a simple surface medication strategy, which enables quantum dots to be covalently attached to the polymerizing PVT chain. After optimization of quantum dots and dye concentration, a maximal light yield of 9275 ph MeV^‐1^ can be obtained in the structured scintillator with the combination of 60% quantum dots/2% FBtF/PVT, which exhibits 11.2% improvement compared with that of bare 2% FBtF/PVT composite (8340 ph MeV^‐1^). When coupled to a commercial PMT, the optimized structured scintillator displays a full‐energy peak with 9.8% energy resolution under radiation from ^137^Cs, which is promising for practical radiation detection. Recently, Brovelli and co‐workers reported an efficient, fast structured scintillator incorporated with CsPbBr_3_ quantum dots.^[^ ^35^ ^]^ Complete energy transfer from embedded particles to dye could be achieved because the highest occupied molecular orbital and lowest unoccupied molecular orbital levels of the dye perfectly match the valence and conduction bands of CsPbBr_3_ quantum dots. Under radiation excitation, the sample exhibits radioluminescence comparable to those of commercial BGO single crystal and Kuraray SCSF‐3HF plastic scintillator, along with fast decay of approximately 3.4 ns.

In addition to the above introduced two types of particle–polymer structured scintillators, another special particle–polymer structured scintillator is the candidate composed of emitting particles and emitting matrix. It has been applied for radiation identification in neutron/gamma mixed field. The working principle involves the combination of particles and polymer matrix with different scintillating properties, including gamma sensitivity, neutron cross‐section, and decay time, which enables pulse shape discrimination. With the help of signal analysis methods, including zero crossing method and charge integration method, the types of the incident particles can be identified as gamma or neutron based on their decay shape characteristics.^[^ ^36^ ^]^ For instance, Rich et al. demonstrated a bulk size neutron detector prototype fabricated by incorporating GS20 Li glass into EJ‐290, a commercially available scintillating PV.^[^ ^37^ ^]^ Combining the different scintillating performances of particles and matrices, GS20/EJ‐290 structured scintillator can provide unique decay shape that can be used to distinguish gamma and neutron under the radiation from a ^252^Cf neutron source. More recently, Frangville and co‐workers reported a novel structured scintillator homogenously embedded with isotopically ^6^Li_2_^10^B_4_O_7_ nanoparticles.^[^ ^38^, ^39^ ^]^ As a result of the varying scintillating performances of thermal neutron sensitive nanoparticles and fast neutron/gamma sensitive matrix, the as‐made sample presented a triple thermal neutron/fast neutron/gamma pulse shape discrimination ability (Figure 4k).

### 2.2 Particle‐Glass Structured Scintillators

Similar to polymer matrix, glass exhibits several attractive properties such as low cost, large volume possibility, thermal stability, and chemical resistance. In addition, its chemical composition can be easily tailored in a wide range, and heavy elements and various efficient luminescent activators can be introduced.^[^ ^40^ ^]^ Nevertheless, due to the amorphous nature, the nonradiative recombination probability is relatively high in glass matrix, resulting in a low light yield. Moreover, the existence of large amounts of defects in glass easily induces generation of color centers when exposed to radiation, leading to poor radiation hardness. Particle–glass structured scintillators embedded with emitting inorganic crystal or quantum dots particles, namely, glass ceramics, combine the advantages of glass matrix and emitting inorganic crystals or quantum dots. It has been regarded as a type of promising structured scintillator.

The design of particle–glass structured scintillators is similar to the particle–polymer structured scintillators. Especially, maintaining transparency is required for particle–glass structured scintillators, and Equation 1 can be employed for this purpose. Similar strategies, including particle size control and refractive index matching, have been utilized for avoiding the transmission loss caused by the scattering of the embedded particles. Particle–glass structured scintillators are generally fabricated through in situ or ex situ method. In the former case, it is fabricated by controlled nucleation and crystal growth of as‐made glass precursor with specific chemical composition. Given the thermodynamically metastable state of glass, atomic rearrangement occurs when heating glass to a certain temperature to remove kinetic barriers. By programmed heat treatment, nuclei can be formed in glass matrix and grow to the designed size. This method has been widely used, and various types of particle‐glass structured scintillator have been reported, which will be detailed introduced below. In the second case, comelting and solution immersion have been proposed. This strategy, despite having a limited number of reports, presents unique advantages in construction of structured scintillators where crystals cannot be successfully precipitated by the first method.^[^ ^41^ ^]^

**Figure 5:**
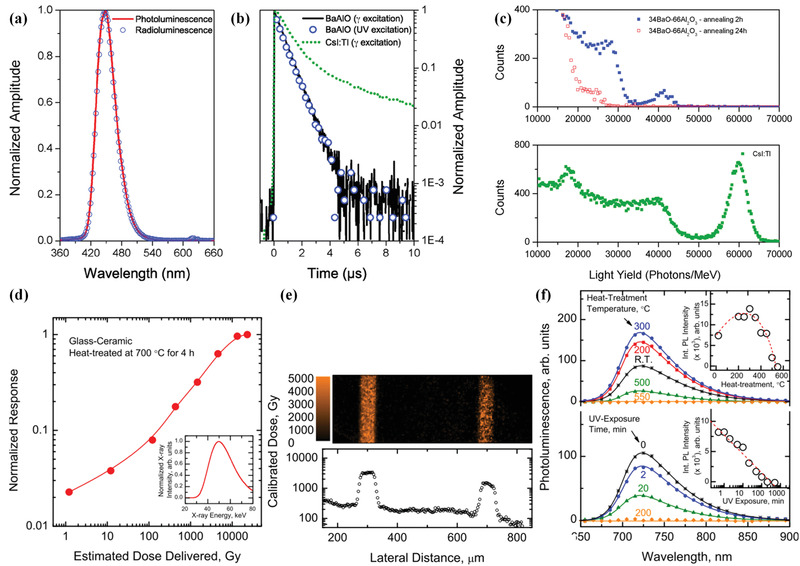
a–c) Fully crystallized BaAl_4_O_7_:Eu^2^^+^ structured scintillator. a) Photoluminescence and radioluminescence spectra. b) Decays under pulse laser excitation at 380 nm and under excitation (662 keV from a ^137^Cs source). c) Pulse height spectra under ^137^Cs irradiation. d–f) Structured scintillators embedded with CaF_2_:Sm^3+^/Sm^2+^ nanocrystals.d) Dose–response curve. e) Dose distribution image of microbeam recorded and digitized using confocal photoluminescence microscopy. f) Photoluminescence spectra recorded during two erasure processes: (top) heat erasure and (bottom) optical erasure. a–c) Reproduced with permission.^[^ ^46^ ^]^ Copyright 2014, Royal Society of Chemistry. d–f) Reproduced with permission.^[^ ^56^ ^]^ Copyright 2014, Wiley‐VCH.

Particle–glass structured scintillators embedded with emitting inorganic crystals can be classified into oxide family and halide family according to the composition of inorganic crystals. Oxide crystals are known for their high density, outstanding stability, and nonhygroscopicity. Via carefully designing the glass composition and heat‐treatment progress, various types of oxide crystals could be precipitated from glass matrix. For instance, structured scintillator embedded with BGO crystals was reported by Polosan and Secu.^[^ ^42^ ^]^ Unexpectedly, the colorless sample was successfully constructed by Yang and co‐workers, based on the rational phase transition control.^[^ ^43^ ^]^ A considerable enhancement of X‐ray excited luminescence could be observed after heat treatment of as‐prepared glass precursor. For neutron detection, Nikitin et al. reported an ^6^Li enriched particle–glass structured scintillator fabricated by comelting LiAlSi_4_O_10_ nanocrystals with glass matrix^[^ ^44^ ^]^ After heat treatment, a high light yield, up to 240% of commercial GS20 glass, could be achieved under excitation with neutron. Moreover, benefiting from tailorable crystallization thermodynamics of glass materials, a particle–glass structured scintillator with a high crystalline volume (even close to 100%) and an excellent optical transmission can be obtained.^[^ ^45^ ^]^ As a typical example, a fully crystallized BaAl_4_O_7_:Eu^2+^ structured scintillator was demonstrated by Dujardin and co‐workers（ **Figure 5**a‐c）.^[^ ^46^ ^]^ After chemical composition and heat‐treatment modification, the optimized sample exhibited a high yield (75% of standard CsI:Tl crystal) and a fast decay of 670 ± 14 ns.

Compared with oxide crystals, halide matrixes are advantageous for improving radiation transition probability due to their low phonon nature. To date, a wide range of halide crystals has been successfully incorporated in glass matrix, including but not limited to CaF_2_,^[^ ^47^ ^]^ SrF_2_,^[^ ^48^ ^]^ LaF_3_,^[^ ^49^ ^]^ GdF_3_,^[^ ^50^ ^]^ CeBr_3_,^[^ ^51^ ^]^ GdBr_3_,^[^ ^51^ ^]^ KLu_2_F_7_,^[^ ^52^ ^]^ and BaLuF_5_.^[^ ^53^ ^]^ As the examples, transparent structured scintillators precipitated with BaGdF_5:_Tb^3+^ and SrGdF_7:_Tb^3+^ were demonstrated by Guo and co‐workers.^[^ ^54^ ^]^ In these materials, Gd element could not only help improve the density and *Z* but also act as a sensitizer for luminescence. Profiting from high radiation stopping power and efficient energy transfer from Gd^3+^ to Tb^3+^, the samples exhibited intense X‐ray excited luminescence with the intensity reaching approximately 140% and 190% of the commercial BGO crystal. The absolute quantum efficiencies of the above samples were calculated as 30.7% and 59.1%. For thermal neutron detection, Kang and co‐workers presented a structured scintillator incorporated with CaF_2_:Eu^2+^ crystals.^[^ ^55^ ^]^ The constructed sample exhibited a performance comparable to the commercially available GS20 glass but with a lower cost. The potential application for radiation dosimetry by using a structured scintillator has been investigated as well. For example, an erasable dosimetry based on CaF_2_:Sm^3+^/Sm^2+^ structured scintillator was proposed, and the application for microbeam radiation cancer therapy was demonstrated by Kasap et al.^[^ ^56^ ^]^ Based on the principle that Sm^3+^ dopant could be reduced to Sm^2+^ upon X‐ray irradiation and reduction ratio shows dose dependence, the sample enabled radiation detection over a wide range from one to several thousands of grays (Figure 5d). Excitingly, a simple processing such as heat‐treatment or UV light exposure could erase the as‐recorded dose information, enabling to reuse of it in practical application. Toward high‐resolution medical imaging, Johnson and co‐workers presented a BaCl_2_:Eu^2+^ structured scintillator.^[^ ^57^ ^]^ Instead of directly emitting luminescence under radiation, BaCl_2_ nanoparticles could store the imaging information by means of trapped electron–hole pairs and release them in the form of photons when irradiated by an external light source. A clear X‐ray image of a mouse leg, whose resolution could reach 110 lp mm^‐1^ at a MTF of 20%, was demonstrated by using a plate sample. Moreover, the introduction of halide crystals in glass matrix could eliminate the unexpected colorization of high concentration cerium doping. A recent study by Zhou et al. demonstrated that colorless scintillating glass doped with 15 mol% cerium can be fabricated via the nano crystallization method.^[^ ^58^ ^]^ Structural and luminescence analysis revealed that the local environment change of dopants from amorphous oxygen‐enriched phase to periodic fluorine‐enriched phase during crystallization is the main physical mechanism of discoloration.

**Figure 6:**
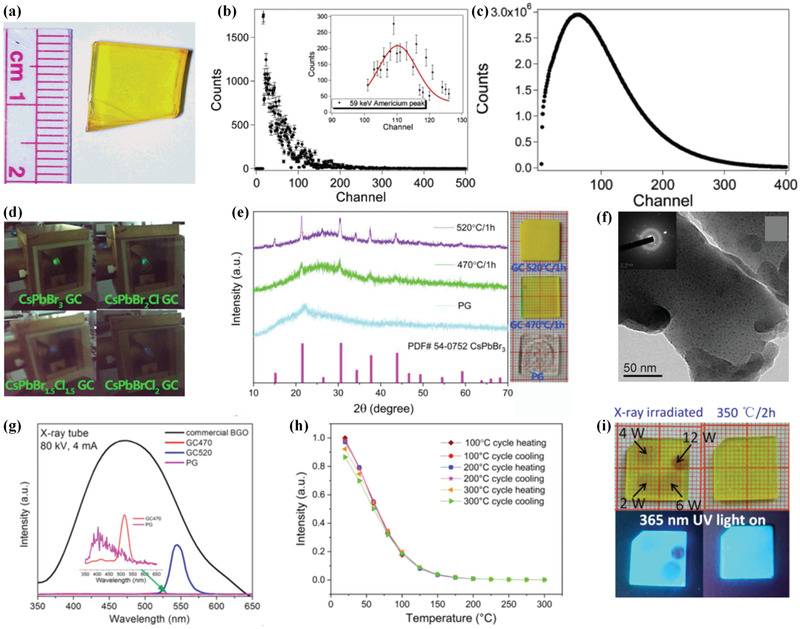
a–c) Particle–glass structured scintillator loaded with CdSe/ZnS quantum dots. a) Photograph. Pulse height spectrum excited by b) alpha particles and c) gamma‐ray. d–i) Structured scintillators precipitated with CsPb(Cl,Br)_3_ perovskite quantum dots. d) Photographs of multicolor radioluminescence under X‐ray irradiation. e) XRD patterns (left) and photographs (right). f) Bright‐field TEM image. g) X‐ray excited luminescence spectrum. h) Temperature‐dependent integrated emission intensity during heating–cooling cycles. i) Photographs and luminescent photos of the structured scintillators after X‐ray irradiation with different powers and then reheating at 350 °C for 2 h. a–c) Reproduced with permission.^[^ ^59^ ^]^ Copyright 2006, American Chemical Society. d–i) Reproduced with permission.^[^ ^60^ ^]^ Copyright 2020, Elsevier.

The other type of particle–glass structured scintillators is the materials embedded with emitting quantum dots. To keep the advantage of high luminescence efficiency of solution‐processed quantum dots, porous glass was employed as the host matrix. Letant and Wang loaded the porous glass with CdSe/ZnS quantum dots by immersing it in the quantum dot solution.^[^ ^59^ ^]^ This method not only allows a homogeneous distribution of quantum dots in glass matrix but also enables achieving high bright luminescence. In **Figure** **6**c, under gamma‐ray radiation from ^241^Am source, the sample exhibited an energy resolution of 15%, which is better than that of standard NaI crystal (30%). The emitting quantum dots were also precipitated inside glass matrix via heat treatment. More recently, Wang and co‐workers reported the fabrication of a structured scintillator incorporated with CsPb(Cl,Br)_3_ perovskite quantum dots.^[^ ^60^ ^]^ The X‐ray excited luminescence test showed that the sample exhibits radioluminescence with the intensity estimated to be approximately 1/18 of the commercial BGO single crystal. Although radiation resistivity was not satisfied because of the soft nature of glass, the sample displayed a unique feature that the high‐power X‐ray‐induced damage can be recovered by thermal annealing (Figure 6i).

**Figure 7:**
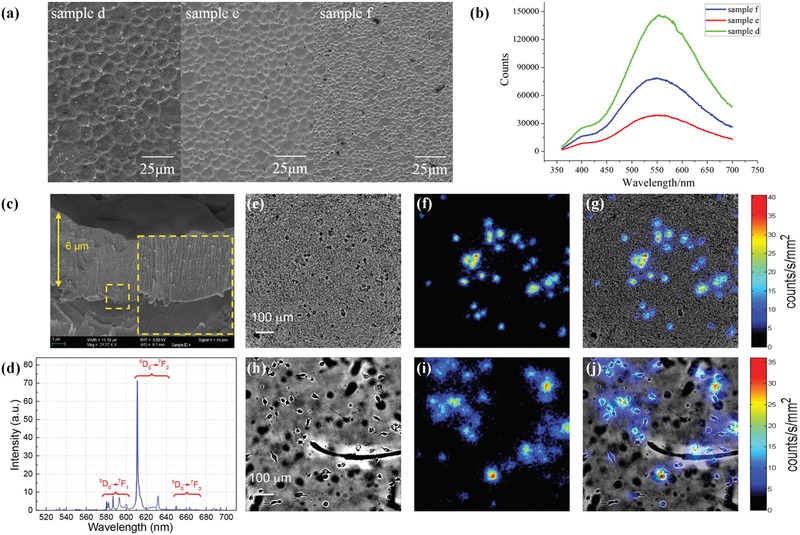
a) SEM images of surface morphology of CsI:Tl thin film and b) photoluminescent spectra with different deposition rate.c‐j) Self‐growth Lu_2_O_3_:Eu array‐derived structured scintillator for high‐resolution radioluminescence microscopy. c) SEM image showing the 6 µm Lu_2_O_3_:Eu film deposited on a sapphire substrate. d) 20 keV X‐ray excited luminescence spectrum. Bright field, radioluminescence, and overlaid micrographs of MDA‐MB‐231 cells imaged using e‐g) Lu_2_O_3_:Eu and h‐j) CdWO_4_. a–b) Reproduced with permission.^[^ ^64^ ^]^ Copyright 2017, Elsevier. c‐j) Reproduced with permission.^[^ ^74^ ^]^ Copyright 2015, Wiley‐VCH.

## 3 Array‐Derived Structured Scintillators

The second category structured scintillator is the array‐derived structured scintillators, which is developed to meet the urgent request of high spatial resolution imaging. For example, X‐ray radiography and X‐ray computed tomography are the most popular techniques for providing a noninvasive approach for in vivo visualization of human tissues and organs, which pose stringent requirements on the spatial resolution of the device. In X‐ray imaging devices, the detector is composed of a scintillator screen and photosensitive receiver arrays, most commonly a charge‐coupled device (CCD). Benefiting from the substantial progress in lithography technique, the CCD matrices can offer millions of pixels per cm^2^. By contrast, classic scintillators are difficult to construct into pixelated panel due to poor mechanical properties. Clearly, novel scintillators that are suitable for the elaboration of pixelated configuration have much room for exploration.^[^ ^4^, ^61^ ^]^ Encouragingly, array‐derived structured scintillator is a promising candidate to achieve a high spatial resolution even with a large thickness. In the following contents, three typical array‐derived structured scintillators, including self‐growth array‐derived structured scintillators, template synthesis of array‐derived structured scintillators, and array‐derived structured scintillators with external coating will be described in detail. The former two structured scintillators are used for high‐resolution imaging. The latter one is mainly applied for improving scintillating efficiency.

### 3.1 Self‐Growth Array‐Derived Structured Scintillators

Two popular strategies, controllable film growth and unidirectional solidification of the eutectic melt, can be used to construct self‐growth array‐derived scintillators. Controllable film growth is derived from the structure zone model, which was proposed by Movachan and Demochishin.^[^ ^62^ ^]^ According to this model, film morphology in vacuum deposition could be controlled by adjusting the ratio of substrate temperature‐to‐deposited material melting point. When the value of the ratio locates in a certain range, columnar structure could be grown as a result of surface diffusion and atomic shadowing. Based on this principle, several scintillating crystals with self‐growth array structure have been successfully constructed.

A representative self‐growth array‐derived structured scintillator based on controllable film growth method is CsI:Tl, which owns a high *Z*(54), a high density (4.53 g cm^‐3^), and a bright radioluminescence (64 000 ph MeV^‐1^), has been widely used as inorganic scintillator in the medicine field. The growth mechanism for the formation of array structure has been investigated. Under appropriate vacuum deposition conditions, the evaporated CsI molecules attach to the substrate and form molecular groups. Then, these molecular groups further capture incident CsI molecules to form a stable island structure. Subsequently, coalescence between these islands structure occurs, and array structure could be observed eventually.^[^ ^63^, ^64^ ^]^ In 1969, Bates first observed the microcolumnar array structure of CsI.^[^ ^65^ ^]^ Subsequently, Fujieda and co‐workers systematically investigated the influence of various deposition parameters including temperature, deposition rate, and subsequent annealing treatment on the formation of array structure.^[^ ^66^ ^]^ Importantly, in 1998, Nagarkar and co‐workers further optimized the structure and successfully constructed a high‐performance array‐derived structured scintillator.^[^ ^67^ ^]^ They improved the quality of the CsI array via postdeposition treatments. This process not only allows filling the defects such as gap between arrays but also helps obtain an additional coating for preventing undesired photon crosstalk. Compared with the untreated reference sample, the newly developed structured scintillator exhibited superior spatial resolution, enabling effective suppression of later spreading light even in a high thick film (≈2000 µm). The crystal orientation of array is also an important factor governing the performance of the structured scintillator. To fabricate the desired structure, Tan and co‐workers investigated the array formation and the related parameter such as deposition rate(**Figure** **7**a,b).^[^ ^64^ ^]^ They revealed that CsI array generally grows along the original crystal orientation. The holes or cracks may affect final orientation, and an excessive deposition rate may cause column collapse or dual crystal orientation.

CsI:Tl array scintillator can be further functionalized for specific detection purposes. More recently, Miller et al. deposited a neutron‐sensitive ^6^LiF layer on the self‐growth array‐derived array layer and demonstrated the application of neutron imaging.^[^ ^68^ ^]^ In this composite array structure, a ^6^LiF layer with a thickness of 60–90 nm was deposited on top of the microcolumnar scintillating film to convert incident neutron into secondary charged particles. The sample displayed a high light yield (≈100 000 ph per neutrons) and neutron/gamma pulse shape discrimination ability with an FOM of 2.8. In the imaging demonstration performed at a neutron imaging beamline at the Oak Ridge National Laboratory, a 3D rendered image of a cockroach specimen was obtained by computed tomography technology, in which minute details of the specimen could be clearly revealed with a high resolution

In addition to CsI, self‐growth array‐derived structured scintillators have been reported with CsBr,^[^ ^69^ ^]^ LaBr_3_,^[^ ^70^ ^]^ LiI,^[^ ^71^ ^]^ Li*_x_* Na_1−_ *_x_* I,^[^ ^72^ ^]^ Lu_2_O_3_,^[^ ^73^, ^74^ ^]^ and ZnO.^[^ ^75^, ^76^ ^]^ For the application of radioluminescence microscopy, Sengupta and co‐workers fabricated Lu_2_O_3_: Eu^3+^ array‐derived structured scintillator with a thickness of 6–10 nm on a sapphire substrate via electron‐beam physical vapor deposition technique.^[^ ^74^ ^]^ The high radiation sensitivity, thinness, and microcolumnar structure of the sample simultaneously helped suppress light spreading, reduce light tracks areas, and finally achieve high spatial resolution imaging. In Figure 7 e‐j, a cell‐based radioluminescence experiment on MDA‐MB‐231 cells showed that the usage of the sample could provide a better‐resolved cell image than that provided by initial used CdWO_4_ scintillators, indicating the potential of single‐cell imaging even in part of confluent cultures or tissue. In another work, Liu and co‐workers synthesized ZnO array‐derived structured scintillator with nanoscale microfeature and characterized its performance.^[^ ^76^ ^]^ Via magnetron sputtering deposition and subsequent hydrothermal reaction method, ZnO nanorods array with an average diameter of 500 nm could be fabricated on quartz substrates with a thickness of 18 µm. The sample exhibited a fast decay time of approximately 3.3 ns and an ultrahigh spatial resolution of 513 lp mm^‐1^ at an MTF of 10%.

The second strategy to construct self‐growth array‐derived structured scintillators is the unidirectional solidification of the eutectic melt. Eutectic is a material composed of at least two coexisting phases in equilibrium, whose properties and microstructure simultaneously depend on individual phase properties, volume fraction, and solidification.^[^ ^77^ ^]^ Depending on these factors, eutectic self‐growth array‐derived structured scintillator could be constructed with two different structures: lamellar structure and rod‐like structure. Lamellar structure is composed of an array of alternating layers, whereas rod‐like structure consists of an array of vertically aligned cylinders in matrix phase. For a given eutectic system, the relationship between solidification rate (*v*) and microstructure interval (*λ*) under steady‐state solidification conditions can be described as *vλ* ^2^ = constants. Additionally, in the scenario of rod‐like structure, solidification rate (*v*) and rod diameter (*a*) could satisfy a similar relationship as well: *va* ^2^ = constants. Guided by these relationships, array structure could be controlled by adjusting solidification rate in fabrication. During eutectic solidification, a solute redistribution occurs because each solid phase rejects the other solute component when growth temperature is smaller than eutectic temperature. The concentration profile in the liquid ahead of the eutectic melt becomes no longer a flat surface, and extensive lateral mixing occurs at the interface. The diffusion flux parallel to the solid–liquid interface deduces the concentration oscillation. Consequently, the concentration gradient across the solid–liquid interface decreases exponentially along the growth direction and leads to the formation of an array structure. Compared with the self‐growth array‐derived structured scintillator with micro columnar feature discussed above, the second phase with a lower refractive index in eutectic could serve as an optical barrier to prevent cross‐talk between adjacent scintillating phase.

Driven by the interest on neutron detection, the early investigation started with the self‐growth array‐derived structured scintillator with lamellar structure made of a scintillating phase and a neutron‐sensitive phase. In 2010, Trojan‐Piegza and co‐workers synthesized CaF_2_/LiF self‐growth array‐derived structured scintillator with different Ca/Li ratios through liquid‐phase consolidation.^[^ ^78^ ^]^ Neutron‐sensitive LiF phase served as a converter, and light emission was completed in CaF_2_:Eu^2+^ phase. Corresponding photopeaks can be clearly observed in the spectra under neutron radiation. Given that the obtained sample exhibited rough lamellar structure and translucency, Fukuda improved the solidification, and a modified CaF_2_/LiF self‐growth array‐derived structured scintillator with a better‐ordered lamellar structure was fabricated via Bridgman method a year later.^[^ ^79^ ^]^ By carefully controlling solidification direction and rate, a fine lamellar structure with an ordered wall shape along the solidification direction could be obtained. To obtain a comprehensive understanding of the effect of rare earth dopants, Masai and co‐workers synthesized and investigated CaF_2_/LiF array‐derived structured scintillators doped with different Eu^2+^ concentrations.^[^ ^80^ ^]^ Characterization presented that excess amounts of Eu^2+^ could not only cause undesired luminescence concentration quenching but also induce irregularity of lamellar structure and reduce transparency. In addition to CaF_2_/LiF, other array‐derived structured scintillators with lamellar feature have been investigated, including SrF_2_/LiF,^[^ ^81^ ^]^ LiSrI_3_/LiI,^[^ ^82^ ^]^ BaCl_2_/LiCl,^[^ ^83^ ^]^ Lu_3_Al_5_O_12_(LuAG)/Al_2_O_3_,^[^ ^84^ ^]^ and CaF_2_/LiBaF_3_/LiF.^[^ ^85^ ^]^ For instance, Yanagida et al. fabricated SrF_2_/LiF structured scintillators via the micro pulling down (µ‐PD) method.^[^ ^81^ ^]^ Under ^252^Cf neutron excitation, the sample exhibited a light yield of 4500–5500 ph n^‐1^ and fast decay time of 120–160 ns.

**Figure 8:**
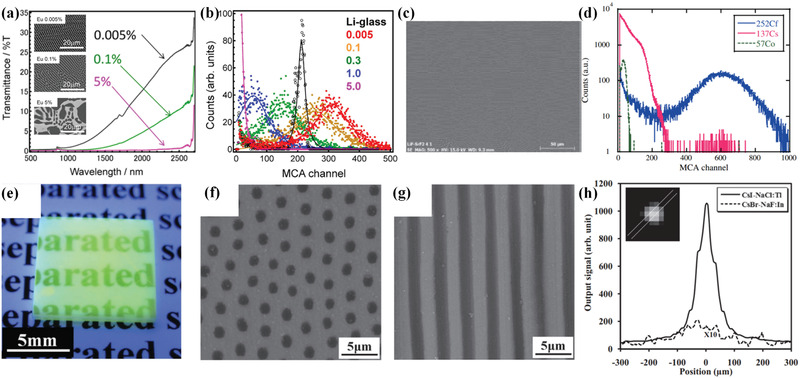
Influence of Eu dopant concentration on a) optical transmittance spectra and b) pulse height spectrum of CaF_2_/LiF structured scintillator. c) SEM images and d) pulse height spectrum of SrF_2_/LiF structured scintillator. e–h) Array‐derived structured scintillators with rod‐like feature. e) Luminescence image under 312 nm UV light. f, g) SEM images of the representative CsI/NaCl sample. h) Line profiles of spot‐excited images for representative CsI/NaCl:Tl (solid line) and CsBr/NaF:In (dotted line) sample. Inset: image of spot‐excited CsI/NaCl:Tl sample. a,b) Reproduced with permission.^[^ ^80^ ^]^ Copyright 2015, Springer Nature. c,d) Reproduced with permission.^[^ ^81^ ^]^ Copyright 2013, Elsevier. e–h) Reproduced with permission.^[^ ^86^ ^]^ Copyright 2012, Wiley‐VCH.

Although remarkable progresses have been performed on array‐derived structured scintillators with a lamellar feature, the rod‐like structure is superior from the perspective of light guiding efficiency and high spatial imaging ability. In 2012, Yasui and co‐workers demonstrated array‐derived structured scintillators with rod‐like feature for the first time (**Figure** **8**e‐h).^[^ ^86^ ^]^ Several alkali halide samples, CsI/NaCl, CsI/RbF, RbI/NaCl, CsBr/NaCl, CsBr/NaF, and RbCl/NaCl, were fabricated through the vertical zone melting method. With the exception of RbCl/NaCl, all samples exhibited efficient light guiding properties. Tl‐doped CsI/NaCl and In‐doped CsBr/NaF samples were selected as representatives for further characterization. Both samples displayed a bright radioluminescence; the former showed a 5.5‐fold higher plane‐excited output and a 62‐fold higher spot‐excited output than those of the latter. Ohashi and co‐workers reported a modified structured scintillator with air rod array because a higher refractive index contrast facilitates the light guiding effect.^[^ ^87^ ^]^ After solidification, the NaCl/CuI and KCl/CuI samples were immersed in water to remove the soluble NaCl and KCl phase from insoluble CuI matrix selectively. Profiting from the large volume fraction, smooth surface, and high refractive index contrast of the optimized sample, X‐ray imaging with a spatial resolution of 10 lp mm^‐1^ could be achieved.

**Figure 9:**
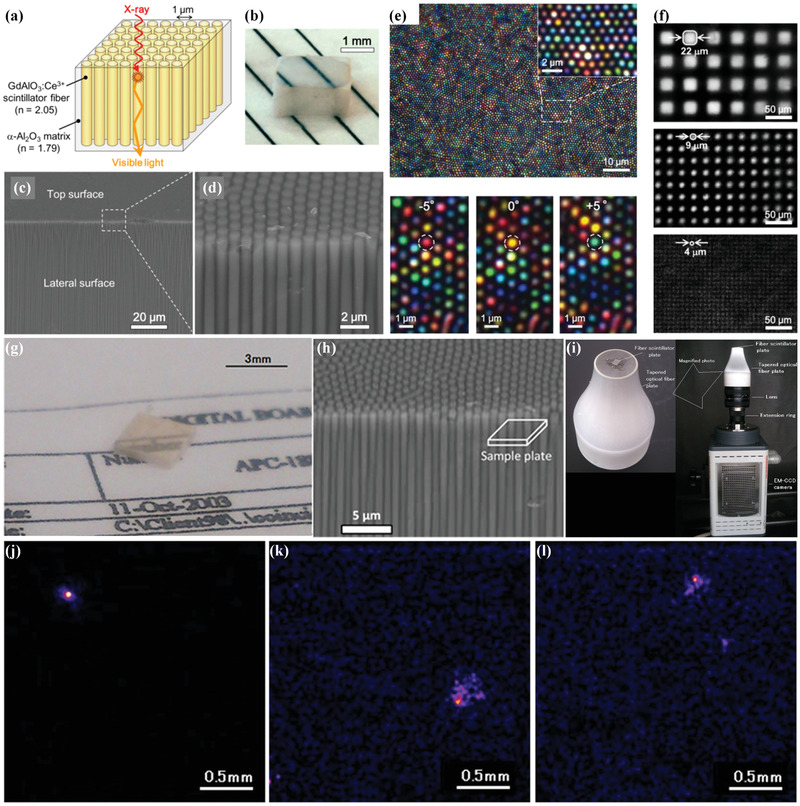
a–f) GdAlO_3_/Al_2_O_3_ array‐derived structured scintillators with rod‐like feature. a) Schematic diagram. b) Photograph. c,d) SEM images. e) Optical transmission microscope images illuminated by a parallel incident white light source of the 1100 µm thick sample (top) and enlarged area with different incident light angles (down). f) Images of gold grating phantoms with aperture‐pitch sizes of 22–43, 9–22, and 4–8.2 µm. g–l) Ultrahigh‐resolution radiation imaging system based on GdAlO_3_/Al_2_O_3_ structured scintillators. g) Photograph. h) SEM images. i) Structure of the radiation imaging system. j–l) Images of typical alpha particles, beta particles, and gamma photons. a–f) Reproduced with permission.^[^ ^88^ ^]^ Copyright 2013, ‐AIP Publishing. g–l) Reproduced with permission.^[^ ^89^ ^]^ Copyright 2018, Springer Nature.

In alkali halide array‐derived structured scintillators with rod‐like feature discussed above, the scintillating phase, which possesses a higher refractive index, constructs as the matrix phase. In this scenario, light‐guiding properties are confined in the matrix phase rather than cylinder array phase while cylindrical optic waveguide is preferred. Based on this principle, Ohashi and co‐workers reported GdAlO_3_/Al_2_O_3_ array‐derived structured scintillator, in which a higher refractive index GdAlO_3_ scintillating phase was constructed as the cylinder array and served for light guiding (**Figure** **9**a–f).^[^ ^88^ ^]^ Via the µ‐PD method, a sample with a hexagonally aligned 680 nm diameter GdAlO_3_:Ce^3+^ cylinder array surrounded by Al_2_O_3_ matrix was fabricated. X‐ray imaging of several gold grating phantoms with a micrometer scale aperture was performed, and even the 4 µm aperture could be resolved. In the following work, Yamamoto et al. demonstrated an ultrahigh‐resolution radiation system fabricated by coupling GdAlO_3_/Al_2_O_3_ array‐derived structured scintillators to a tapered optical fiber plate and CCD camera (Figure 9g–l).^[^ ^89^ ^]^ Imaging performance was characterized by using different radiation sources, including alpha particles, beta particles, and gamma ray. Under alpha particle excitation, an image with a spatial resolution of approximately 25 µm could be obtained, whereas under beta particle and gamma‐ray excitation, the images exhibited various shapes, clearly showing the trajectories of incident beta particle or secondary electron created by gamma ray/material interaction (Figure 9j‐l).

### 3.2 Template Synthesis of Array‐Derived Structured Scintillators

**Figure 10:**
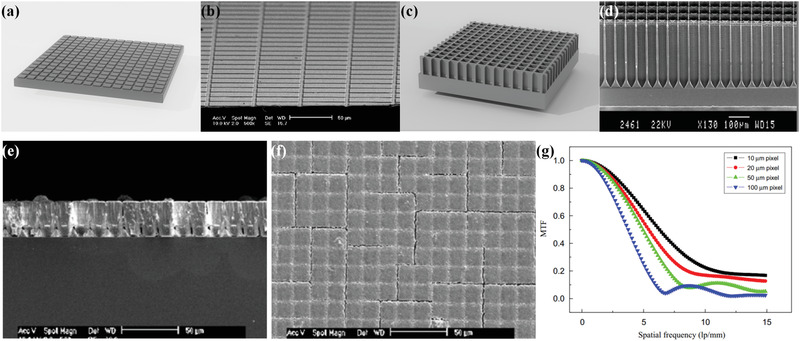
Schematic diagram and SEM image of a,b) template with surface pattern and c,d) template with pore array. e–g) CsI(Tl) structured scintillator synthesized with a surface patterned template. e,f) SEM images of the structured scintillator with 10 µm column interval. g) Measured MTF curves of the scintillators of various column intervals. b,e–g) Reproduced with permission.^[^ ^91^ ^]^ Copyright 2007, Elsevier. d) Reproduced with permission.^[^ ^93^ ^]^ Copyright 2008, Wiley‐VCH.

Owing to the lack of an effective optical isolation between adjacent columns, further development of the controllable film growth strategy to fabricate array‐derived structured scintillators is hindered by the cross‐talk problem. A large proportion of scintillation light may escape through microcolumn boundaries and enter adjacent microcolumns, thus deteriorating overall imaging resolution. To match the request of practical applications, a modified strategy derived from controllable film growth was developed, namely, template synthesis. By utilizing a special template, self‐growth array‐derived structured scintillators with microcolumnar feature could be reshaped into array with the designed geometry, size, and more importantly, optical barriers in inter‐pixel gaps. To date, two kinds of templates have been widely developed for fabrication of array‐derived structured scintillators: template with surface pattern (**Figure** **10**a) and template with pore array (Figure 10c).

The surface pattern with microcolumnar array structure could be formed on the template surface via photolithography or laser processing. In the following deposition, evaporated particles are not likely to enter gaps between each pattern unit due to the shadowing effect.^[^ ^90^ ^]^ Instead, those particles nucleate directly and grow along with the predesigned patterns, presenting a pathway to fabricate array‐derived structured scintillator via utilizing a template with a surface pattern. To meet the basic requirement of the shadowing effect, columnar interval and column height need to be carefully controlled. Normally, a column interval of less than 1 µm and a column height of 1.5 to 2 µm are requested. For example, Kim and co‐workers demonstrated X‐ray imaging detectors basing on CsI:Tl structured scintillators synthesized with a surface patterned template.^[^ ^91^ ^]^ In their experiments, square column array hump patterns with different column intervals (10, 20, 50, and 100 µm) were fabricated on silicon substrate via photolithography. After thermal evaporation of CsI, all four samples reproduced the surface pattern features of the designed substrate and showed remarkable improvement of spatial resolution and signal‐to‐noise ratio compared with nonstructured scintillator. To improve imaging resolution further, Yao and co‐workers modified the template and fabricated a structured scintillator with finer array.^[^ ^92^ ^]^ An array with a period of 3 µm was patterned on the template surface, and a spatial resolution of approximately 18 lp mm^‐1^ at a MTF of 10% could be obtained with the optimized sample.

The template with pore array can also be used to the constructed structured scintillator. Via deep‐reactive‐ion‐etching or electrochemical etching, an aligned and ordered deep pore array with a specific shape can be formed in the substrate. Each pore serves as a single light guide, and the pore wall works as an intrinsic optical barrier to suppress crosstalk between adjacent units. Moreover, oxidation of silicon pore wall or deposition a metal layer through methods such as atomic layer deposition could reduce the refractive index of pore wall, further improving light guiding efficiency. After substrate preparation, scintillator powders or slurry are filled into the pore array and subsequently heated. Finally, array‐derived structured scintillators could be successfully fabricated in a silicon substrate. Array‐derived structured scintillator synthesized by template with pore array was first demonstrated by Kleimann and co‐workers in 1999.^[^ ^94^ ^]^ A template with a pore size of 45 µm and a pore depth of 185 µm was prepared via deep‐reactive‐ion‐etching. Based on this template, a structured scintillator with an imaging resolution of 10 lp mm^‐1^ at an MTF of 14% and a light output reaching 23% of bulk CsI crystal could be obtained. Imaging performance is simultaneously affected by various parameters, including pore size, pore wall thickness, pore shapes, and array structure. Generally, a small pore size and a thin wall seem to facilitate a high spatial resolution. Based on this principle, Hormozan et al. reported a modified CsI structured scintillator by using a template with a more compact pore array.^[^ ^95^ ^]^ Combining conventional optical lithography and deep reactive ion etching, a template with a small pitch (4 µm) and a wall thickness (≈1 µm) was prepared. To increase refractive index contrast, a 250 nm SiO_2_ layer was formed on the pore wall by dry oxidation after pore etching, and the optimized sample showed a spatial resolution of 100 cy mm^‐1^. However, pore wall thickness also exhibits the optimal value for effectively preventing escaping light to enter surrounded column, and several micrometers are usually required. Moreover, the difficulty in fabricating a template with an ultrasmall pore array and fully filling ratio in those extreme small pores should be considered. To optimize pore wall thickness, Chen et al. simulated and investigated its influence via Geant4 Monte Carlo simulation code.^[^ ^96^ ^]^ They found that decreasing thickness to less than 0.5 µm leads to a sharp reduction of spatial resolution, which is mainly due to the suppressed ability of pore walls to prevent cross‐talking.

**Figure 11:**
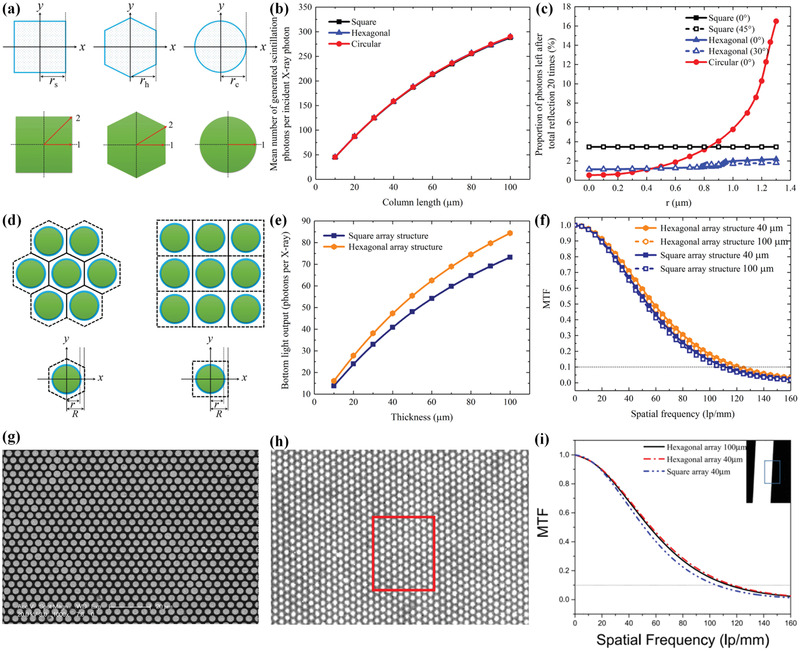
a–f) Influence of pore shape and pore array arrangement on performance of CsI structured scintillator studied via simulation. a) Schematic diagram of different pore shapes and their effect on b) X‐ray absorptions and c) proportion of photons left after total reflection. d) Schematic diagram of different pore array arrangements and their effect on e) X‐ray absorptions and f) MTFs. g–i) Compact CsI structured scintillator with hexagonal array arrangement. g) SEM image. h) X‐ray image. i) MTF curves of fabricated CsI structured scintillator. Inset: X‐ray image of a lead slit. a–f) Reproduced with permission.^[^ ^97^ ^]^ Copyright 2018, Springer Nature. g–i) Reproduced with permission.^[^ ^98^ ^]^ Copyright 2018, Elsevier.

In addition to pore wall thickness, investigations on other parameters, including pore shape and pore array arrangement, have been performed. Similar to the above research focusing on pore wall thickness, Chen et al. simulated the effect of pore shape and pore array arrangement by using Geant4 Monte Carlo simulation code in another work (**Figure** **11**a–f).^[^ ^97^ ^]^ The scintillating performances of structured scintillators with three different shapes (square, hexagonal, and circular) and two pore array arrangements (square and hexagonal) were investigated and discussed in detail. The results indicated that pore shapes have almost no influence on the mean number of the generated scintillation photons per incident X‐ray photon but strongly affect the proportion of photons left after total reflection and therefore, the light output. When incident positions depart from the centers of the cross‐sections, the corresponding proportion for circular shape increases exponentially, whereas that for hexagonal and square varies slightly, indicating a better light guiding efficiency for the circular pore shape. For the effect of pore array arrangement, hexagonal array arrangement displays a more compact feature compared with traditional square array arrangement. With the same pore shape, size, and thickness, the light output, spatial resolution, and detective quantum efficiency of a hexagonal array arranged sample are all higher than those of a square array arranged sample. The above results indicate that an array‐derived structured scintillator with circular shaped and hexagonal array arrangement might be the optimal solution. In another work performed by Gu and co‐workers, hexagonal and square arranged CsI array‐derived structured scintillator with a pore diameter of 3 µm and a pore pitch of 1 µm were elaborated.^[^ ^98^ ^]^ Benefiting from the compact feature of hexagonal array arrangement and fine pore array structure, the hexagonal arranged candidate displayed a high spatial resolution of approximately 115 lp mm^‐1^ (Figure 11i).

In addition to array‐derived structured scintillators designed for high‐resolution imaging, another prosing candidate, which is also known as metascintillators, has been proposed to address the requirement of ultrafast gamma detectors.^[^ ^99^ ^]^ This special kind of array‐derived structured scintillators is composed of multiple high Z material layers such as traditional inorganic scintillating crystals, which provide efficient radiation stopping power and energy resolution, and fast photon emitters layers, which improve timing performance. Via rationally constructing the array structure to make the layers thin enough, part of the recoil electrons from the high Z materials layers can enter the emitters layers, enabling a faster decay and facilitating a superior coincidence timing resolution. The first experimental proof of concept was demonstrated by Turtos and co‐workers in 2019.^[^ ^100^ ^]^ Commercially available BC‐422 fast plastic scintillator along with BGO or LYSO crystals were selected, machined into layer with a thickness of several hundred micrometers, and bound together. Geant4‐based Monte Carlo simulations revealed that 14.8% energy was shared by the two layers for the BGO‐BC422 sample and 12.7% for the LYSO‐BC422 sample. In the performed experiment, a coincidence timing resolution of 95 ps could be obtained with BGO‐BC422 compared with 195 ps of standard BGO crystal while the LYSO‐BC422 samples showed a coincidence timing resolution of 55 ps compared with 83 ps of the standard LYSO crystal. These values are highly promising at the early stage, and this novel type of structured scintillators can open a door to a new generation of ultrafast scintillator.

### 3.3 Array‐Derived Structured Scintillators with External Coating

In addition to improving image resolution, array‐derived structured scintillators are developed for enhancing light extraction efficiency and the resultant detection performance. For a common radiation detection system, detecting efficiency is simultaneously dependent on the internal quantum efficiency of scintillator and light extraction efficiency. Benefiting from the advanced material synthesis technique, the internal quantum efficiency of numerous scintillators has almost reached the limit. In stark contrast, the light extraction efficiency in the detection system is still at a relatively low level. This finding is mainly associated with the large refractive index contrast between scintillator and optical glue or grease, which leads to a relatively small total refraction critical angle at the scintillator/glue (grease) interface. As a result, a large proportion of light would be trapped inside the scintillator and finally leak at the edge or be absorbed by the scintillator. To collect the light generated by the scintillators fully, a strategy of fabricating array‐derived structured scintillators with external coating was proposed. In principle, the array structure on top of scintillator, namely, the photonic crystal structure, could modify the optical modes and light propagation habit, thus enhancing the light extraction efficiency. Given the difficulty in directly processing the scintillator surface, a structured layer, commonly Si_3_N_4_ and TiO_2_, has been deposited on top of the scintillator via external coating.^[^ ^101^ ^]^ Lithography technologies, including electron‐beam lithography, soft‐X‐ray interference lithography, and nano imprint lithography are commonly employed. The self‐assembly method has also been developed to construct array‐derived structured scintillator with external coating.

Electron‐beam lithography involves using a focused electron beam to create the designed pattern on the surface of scintillator. An initial exploration was made by Knapitsch and co‐workers based on LYSO array‐derived structured scintillator with external coating.^[^ ^102^ ^]^ After depositing a Si_3_N_4_ layer on top of LYSO crystal, array structure consisting of hexagonally arranged air holes was lithographed on the external coating through electron‐beam lithography. However, due to the irregular deposition of the electro‐sensitive resist, the effect area of external coating was only limited to micrometer scale, and no further characterization was performed. In the following work demonstrated on LSO crystal, they modified the lithography and successfully constructed array‐derived structured scintillator with a millimeter‐scale external coating.^[^ ^103^ ^]^ Two types of array structures, holes and pillars with different parameters and geometry, were lithographed on the Si_3_N_4_ auxiliary layer. Compared with unstructured reference, structured scintillator showed a 20%–60% improvement in light yield. Electron‐beam lithography is a good method for constructing array‐derived structured scintillator with external coating for prototype demonstration due to its flexibility, but it is high cost and extremely time consuming for large‐area production. To achieve fast, cost‐effective, and large‐area production, two other methods, soft‐X‐ray interference lithography and nanoimprint lithography, were developed.

Soft‐X‐ray interference lithography is based on the interference of two or more coherent X‐ray beams. It enables producing a large‐area array structure that is similar to the interference wave form in a short time. For example, Liu and co‐workers reported a BGO structured scintillator with external coating.^[^ ^104^ ^]^ BGO crystal was first spin‐coated with polymethyl‐methacrylate (PMMA) photoresist and then exposed to soft‐X‐ray through a mask to form an interference pattern. Subsequently, a conformal TiO_2_ layer was deposited on the as‐made pattern via atomic layer deposition to minimize the low refractive index effect of PMMA. The size of array structure can reach 5.6 × 5.6 mm^2^, and the structured sample showed a 95.1% output enhancement compared with the plane reference. In another work performed by Wang and co‐workers, an external coating with the configuration of circular holes array and a size of 8×8 mm^2^ was fabricated on top of a Y_3_Al_5_G_12_:Ce (YAG) structured scintillator.^[^ ^105^ ^]^ In the case of air coupling, the light output of the sample was ≈2.8 times higher than that of the unstructured reference.

**Figure 12:**
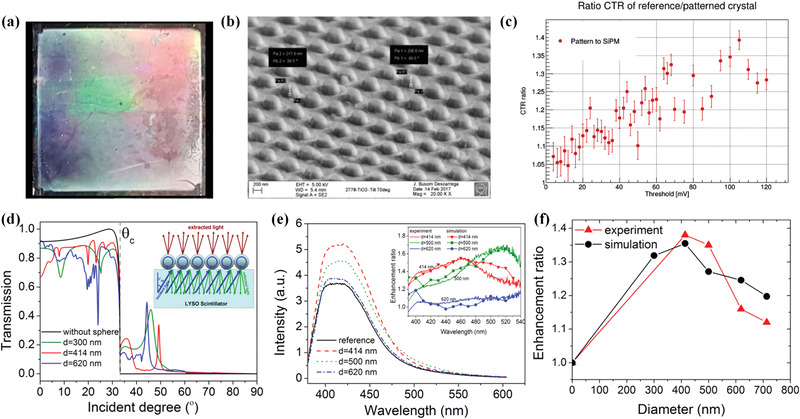
a–c) Structured scintillator with a pillar array structured coating fabricated by nano imprint lithography. a) Photo of the nano imprinted surface showing the typical iridescent diffraction effects. b) SEM image of sample tilted by 70 degrees with 20k magnification. c) . Ratio of the coincidence time resolution obtained for the patterned crystal and the reference crystal. d‐f) LYSO structured scintillator with external coating. d) Simulated transmission at 415 nm as a function of incident angle. e) Photoluminescence spectra in the normal direction. Inset: enhancement ratio with respect to the reference sample. f) Experimental and simulated enhancement ratio of light extraction at 415 nm emission.a–c) Reproduced with permission.^[^ ^107^ ^]^ Copyright 2019, Elsevier. d‐f) Reproduced with permission.^[^ ^108^ ^]^ Copyright 2013, AIP Publishing.

Nano imprint lithography, which is based on pressing a stamp with the negative of the desired pattern into the material surface, has also been proven as a fast, cost‐effective method for fabricating array‐derived structured scintillators. By repeating the stamping, a large area of array structure could be imprinted on the external coating layer even when using a small template. For instance, Singh et al. reported three different array‐derived structured scintillators with external coating fabricated by nanoimprint lithography.^[^ ^106^ ^]^ External coating consisting of a square packed array of nanocones was directly lithographed on the auxiliary layer of Lu_1.8_Y_0.2_SiO_5_:Ce (LYSO) crystal and (Gd, Y)_3_(Al, Ga)_5_O_12_:Ce (GYGAG) ceramic. Both structured scintillators showed substantial enhancements in light extraction efficiency (20% for LYSO and 50% for GYGAG) and energy resolution (18% for LYSO and 9% for GYGAG). Unexpectedly, in the detector composed of hygroscopic SrI_2_ crystal and quartz window, an array structure could be printed on the quartz window, and it helped obtain a 42% gain in light extraction efficiency and a 15% gain in energy resolution Recently, an LYSO array‐derived structured scintillator with a pillar array structured coating was reported by Pots and co‐workers (**Figure** **12**a–c).^[^ ^107^ ^]^ Compared with the plane reference, the sample displayed 50%, 10%, and 20% improvements in light yield, energy resolution, and coincidence time resolution, respectively.

Self‐assembly is a process in which the pre‐existing components of a disorder system self‐organize into an ordered structure duo to the special local interactions among those components without external direction. It has been widely used for constructing nanostructures for electronic and biological applications. Liu and co‐workers applied it to construct an array‐derived structured scintillator with external coating via self‐assembling PS spheres with different diameters on top of LYSO crystal (Figure 12d‐f).^[^ ^108^ ^]^ Compared with the unstructured reference, the structured scintillator exhibited a 38% enhancement in light yield. In the following work, they further improved the performance of the LYSO array‐derived structured scintillator with by conformally depositing a TiO_2_ layer on the PS spheres because the refractive index of PS (1.59) is relatively low compared with LYSO crystal.^[^ ^109^ ^]^ The high refractive index of TiO_2_ facilitated the scintillation light coupling between scintillator matrix and external coating, and a 149% increasement of light yield could be obtained with the modified sample. In another work reported by Yi et al., LSO array‐derived structured scintillator with bioinspired moth‐eye structure as external coating was fabricated by the self‐assembly method combined with the recitative ion etching method.^[^ ^110^ ^]^ Differently, 400 nm SiO_2_ particles were deposited on the Si_3_N_4_ auxiliary layer as a mask layer, and subsequent etching led to the formation of the desired structure. Under X‐ray excitation, the luminescence peak intensity at 420 nm of the structured scintillator increased by approximately 1.75 times compared with the unstructured reference. Recently, LYSO structured scintillator with a monolayer of micro lens array was demonstrated by Liu and co‐workers.^[^ ^111^ ^]^ PS microspheres with different diameters (1, 2, and 4.5 µm) were dispersed on silicon wafer to form a monolayer and then transferred to the surface of LYSO crystal. Array size can reach 10 × 20 mm^2^, and a 3.26 times enhancement of radioluminescence with an emission angle of 45° can be observed.

## 4 Fiber‐Derived Structured Scintillators

The third type of structured scintillators is fiber‐derived structured scintillators, which combines the function of scintillation light emission and transmission with its unique fiber configuration, arousing great attention. On the one hand, the 1D linear extension feature of fiber configuration enables real‐time and distributed radiation monitoring. On the other hand, the imaging plate for high‐resolution imaging can be easily constructed by aligning fiber‐derived structured scintillators. In addition, the flexibility of fiber‐derived structured scintillators offers a high degree of geometrical adaptability and compactness in detector design and construction.^[^ ^112^ ^]^ Attracted by these advantages, various kinds of scintillating fibers were designed, fabricated, and studied in the past decades. They could be mainly divided into three groups according to their component materials: glass, crystal, and polymer fiber‐derived structured scintillators.

### 4.1 Glass Fiber‐Derived Structured Scintillators

Benefiting from the easily shaping feature of glass, glass fiber‐derived structured scintillators have been widely studied with decades of history for radiation monitoring. Nearly 40 years ago, findings revealed that silica fiber could be luminescent after exposure to radiation due to the presence of impurities and native or radiation‐induced defects. Fiber dosimetry can be constructed because radioluminescence intensity is proportional to dose rate.^[^ ^113^ ^]^ For example, Darafsheh et al. reported a proton therapy dosimetry based on a bare silica fiber‐derived structured scintillator.^[^ ^114^ ^]^ Under excitations from 100 MeV proton beam, dosimetry showed two distinct peaks at 460 and 650 nm. Unexpectedly, the emission intensity of 650 nm peak was dependent on the dose rate that can be confirmed by reference data calibrated by a standard multilayer ion chamber device. Given that radioluminescence from impurities and defects is relatively weak, rare earth activators such as Ce^3+^ and Eu^3+^ have been selected as active dopants to enhance light output. For instance, Vedda et al. fabricated Ce^3+^‐doped silica fiber‐derived structured scintillator and further demonstrated a radioluminescence dosimetry system.^[^ ^115^ ^]^ Ce^3+^‐doped silica glass powder was synthesized by the sol‐gel method and filled into a quartz tube for fiber drawing. Under X‐ray and gamma‐ray excitation from X‐ray tube and ^22^Na radioisotope source, the sample emitted a strong, fast luminescence that is similar to bulk Ce^3+^‐doped silica glass. Furthermore, a prototype dosimetry system was constructed by splicing the structured scintillator to commercial optical fiber, and the characterization results showed that dose rates below 10^−3^ mGy s^‐1^ could be monitored with a fine linearity. In another work performed by Carrara and co‐workers, a dosimeter based on Ce^3+^‐doped silica fiber‐derived structured scintillator was constructed for the application of high dose rate brachytherapy.^[^ ^116^ ^]^ In the performed measurement, the dosimeter showed high intra session reproducibility, fine linearity, and independence of energy and dose rate. In practical application, especially in high‐energy physics, structured scintillator is required to resist radiation damage and maintain stability for long‐term use. Moretti and co‐workers investigated the radiation hardness of Ce^3+^‐doped silica fiber‐derived structured scintillator.^[^ ^117^ ^]^ They revealed that radiation resistance of structured scintillator can be enhanced by a silica network rearrangement during fiber drawing. Occurrence of radiation‐induced defects is related to the presence of dopants, and a low concentration of Ce is beneficial to the improvement of radiation hardness. In another work, the radiation hardness of Pr^3+^‐doped silica fiber‐derived structured scintillator was studied by Fasoli and co‐workers, and a similar conclusion that fiber drawing could enhance the Pr^3+^‐doped silica fiber radiation hardness could be drawn.^[^ ^118^ ^]^

**Figure 13:**
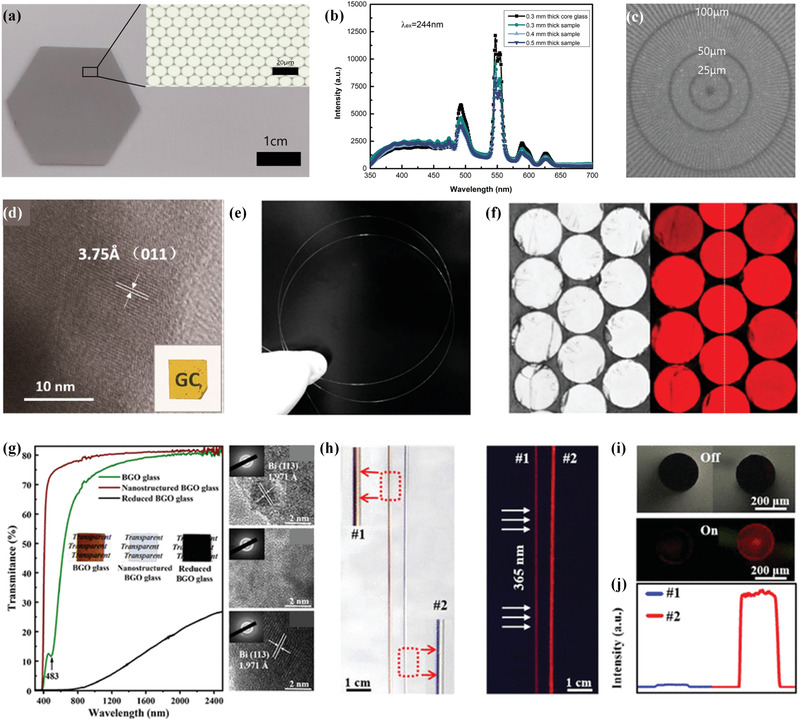
a–c) Neutron imaging with Tb^3+^/Ce^3+^ codoped Gd_2_O_3_ glass fiber‐derived structured scintillator. a) Photograph. b) Photoluminescence spectra of 0.3 mm thick core glass and three structured scintillators with differing thicknesses. c) Cold neutron image of the PSI Siemens Star with the 0.3 mm thick structured scintillators cold neutron imaging system. d–f) Glass fiber‐derived structured scintillator embedded with GdTaO_4_ nanocrystals. d) HRTEM image. Inset: photograph of the parent bulk glass sample. e) Photograph of glass fiber‐derived structured scintillator. f) Optical microscope image of fiber array under natural light (left) and ultraviolet light (right). g–j) Glass fiber‐derived structured scintillator embedded with Bi_2_GeO_5_ nanocrystals. g) Transmittance spectra (left) and TEM images (right). h) Optical microscopy image under natural light and 365 nm UV light. i) Fiber cross‐section with and without 365 nm light excitation. j) Luminescence intensity distribution at the end of fiber. a‐c) Reproduced with permission.^[^ ^121^ ^]^ Copyright 2020, Elsevier. d–f) Reproduced with permission.^[^ ^123^ ^]^ Copyright 2017, American Chemical Society. g–j) Reproduced with permission.^[^ ^43^ ^]^ Copyright 2020, American Chemical Society.

In addition to silica fiber‐derived structured scintillators, multicomponent fiber‐derived structured scintillators have been developed due to their fascinating tunability in chemical composition, especially for the application of neutron detection and imaging. In 2003, for areal density measurements of inertial confinement fusion capsules, Izumi and co‐workers developed a neutron detector basing on Li glass fiber‐derived structured scintillators.^[^ ^119^ ^]^ A highly segmented detector consisting of 3200 segments of 600 µm square fiber‐derived structured scintillators was constructed and showed an intrinsic detection efficiency of 0.74% for 2.45 MeV neutrons and 1.9% for scattered neutrons (271–612 keV). More recently, Moore and co‐workers demonstrated an imaging faceplate based on Li glass fiber‐derived structured scintillator.^[^ ^120^ ^]^ A ^6^Li‐enriched glass rod was first inserted into a commercial N‐KF9 cladding tube and then drawn into single‐core fibers. Subsequently, as‐made single‐core fibers were stacked as a hexagonal array and secondary drawn into multicore fibers. A faceplate consisting of 100 multicore fiber‐derived structured scintillators was constructed, enabling neutron radiographs with a spatial resolution of 16.1 ± 0.5 µm. In another work, Zhang et al. fabricated an imaging array based on Tb^3+^/Ce^3+^ codoped Gd_2_O_3_ glass fiber‐derived structured scintillator for the application of cold neutron imaging (**Figure** **13**a‐c).^[^ ^121^ ^]^ Images of Siemens Stars were taken with samples of different thicknesses, and the best spatial resolution was estimated to be approximately 50 µm. A spatial resolution of approximately 7 µm can be achieved by upgrading the optical system because such a spatial resolution is mainly limited by the optical readout system.

In applications of X‐ray or gamma‐ray detection and imaging, multicomponent glass fiber‐derived structured scintillators doped with heavy elements are preferred. In early work, two kinds of imaging plates based on Tb‐doped multicomponent fiber‐derived structured scintillators were fabricated and characterized by Pavan and co‐workers.^[^ ^122^ ^]^ Compared with the typical phosphor screen, both plates showed a higher spatial resolution and a longer X‐ray attenuation length. Given that glass scintillator normally suffers from low light yield, the strategy of introducing particles into the glass matrix introduced in Section 2 could also be extended to construct glass fiber‐derived structured scintillator. For example, Zhou and co‐workers demonstrated a fiber‐derived structured scintillator embedded with GdTaO_4_ nanocrystals (Figure 13d–f).^[^ ^123^ ^]^ A novel strategy of in situ phase transition control in glass was introduced to activate dopants with a high efficiency and control the crystal growth to maintain transparency simultaneously. Benefiting from the dual energy transfer from Gd^3+^ and TaO_4_^3−^ to Eu^3+^, a bright luminescence could be observed under X‐ray excitation. In another work, Zhou and Yang and co‐workers first reported the successful construction of colorless bismuth germanate BGO bulk glass and glass fiber‐derived structured scintillator (Figure 13g–j).^[^ ^43^ ^]^ Glass relaxation was processed via heat treatment to dissolve unexpected Bi nanoparticles, which are major causes of undesired coloring problem. Compared with the reference sample, the structured scintillator showed approximately 6.28 times enhancement in optical transmittance and approximately 19.48 times enhancement in X‐ray‐induced luminescence intensity.

### 4.2 Crystal Fiber‐Derived Structured Scintillators

**Figure 14:**
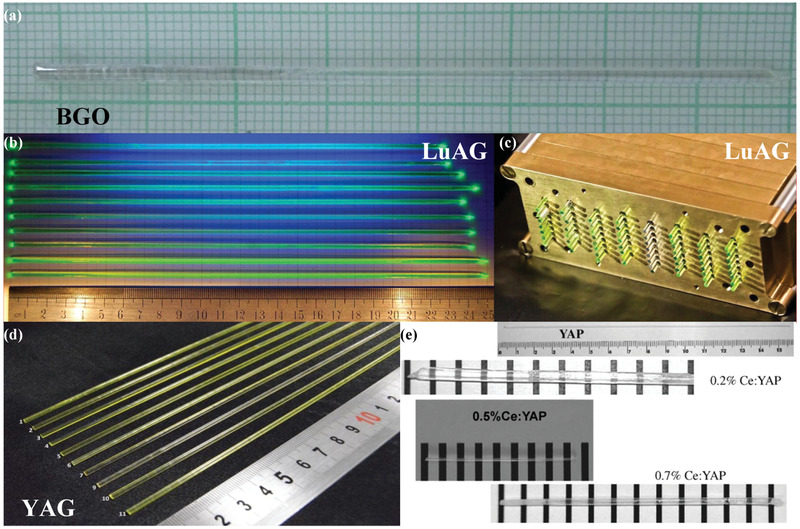
Photographs of representative crystal fiber‐derived structured scintillators. a) BGO. b) LuAG and c) the related calorimeter prototype. d) YAG. e) YAP. a) Reproduced with permission.^[^ ^127^ ^]^ Copyright 2019, Elsevier. b) Reproduced with permission.^[^ ^129^ ^]^ Copyright 2016, Elsevier. c) Reproduced with permission.^[^ ^130^ ^]^ Copyright 2016, IOP Publishing. d) Reproduced with permission.^[^ ^133^ ^]^ Copyright 2019, Royal Society of Chemistry. e) Reproduced with permission.^[^ ^134^ ^]^ Copyright 2007, Elsevier.

Single crystal fibers have also attracted much attention for fiber‐derived structured scintillator due to their outstanding mechanical, optical, and scintillation properties. Driven by the development of the laser‐heated pedestal growth (LHPG) technique and µ‐PD technique, some remarkable progress has been made in the construction of crystal fiber‐derived structured scintillators in recent years.^[^ ^124^ ^]^ The LHPG technique uses a laser, commonly CO_2_ laser, as a heat source to melt the source rod and then slowly pulls up the immersed seed crystal from the melt. The potential contamination from the crucible could be well prevented because no crucible is required in the whole process. The µ‐PD technique is based on the crucible with one or more microchannels at the bottom. The melt is pulled down through the microchannels at a controlled speed to draw fiber. In contrast to LHPG, the crucible plays a critical role in the fiber drawing, whose microchannel parameters can be adjusted to tune fiber cross‐section shape and diameters. Via the above two novel techniques, several popular scintillating crystals have been successfully constructed into fiber structure, including BGO, Lu_3_Al_5_G_12_ (LuAG), YAG, and YAlO_3_ (YAP), as shown in **Figure** **14**. In the following contents, several examples are selected and further discussed.

BGO crystal is a well‐established scintillator, accounting for nearly half of the market of nuclear medical imaging. In early work, Fu and Ozoe fabricated BGO fiber‐derived structured scintillator with a diameter of approximately 0.18–5.2 mm and a length of approximately 35–318 mm via a modified floating zone device.^[^ ^125^ ^]^ To improve luminescence intensity and radiation damage resistance, rare earth elements have been selected as dopants. Shim grew the Eu^3+^‐doped BGO fiber‐derived structured scintillator with a fiber diameter and a length of approximately 1.2 and 33 mm through the µ‐PD method in 2003, respectively.^[^ ^126^ ^]^ Owing to the nonradiative energy transfer from Bi^3+^ to Eu^3+^, BGO intrinsic luminescence is partly quenched, and the emission of Eu^3+^ can be clearly detected. In the following work reported by Xu recently, a Eu^3+^‐doped BGO fiber‐derived structured scintillator with a diameter of 2 mm and a length of 83 mm was fabricated via the µ‐PD method.^[^ ^127^ ^]^ To fabricate BGO fiber‐derived structured scintillator with micrometer‐scale diameter, which is mainly hindered by the serious evaporation problem of Bi_2_O_3_, Chani developed an evaporation‐induced diameter control strategy.^[^ ^128^ ^]^ Through Bi_2_O_3_ self‐flux evaporation compensation, BGO fiber‐derived structured scintillators with self‐control diameter of 50–300 µm were successfully fabricated. In addition to BGO crystal, LuAG crystal is known for its high density, *Z* number, and bright luminescence for the detection of hard X‐ray and gamma ray. Kononets and co‐workers constructed Ce^3+^‐doped LuAG fiber‐derived structured scintillators via the µ‐PD method for application of dual readout calorimetry.^[^ ^129^ ^]^ Fiber diameter and length were approximately 2.2 and 25 mm, respectively. In the following work, a calorimeter prototype consisting of 64 LuAG fiber‐derived structured scintillators was constructed and tested at the Fermilab Test Beam Facility, showing the potential of practical application.^[^ ^130^ ^]^ As to YAG_,_ another well‐known garnet scintillating crystal, Kurlov et al. fabricated it into a fiber‐derived structured scintillator in 2005.^[^ ^131^ ^]^ Via the internal crystallization method, fibers with a diameter of 50–100 µm and a length of 100 mm could be obtained. In another work performed by Lebbou and co‐workers, YAG fiber‐derived structured scintillator with a diameter of 80 µm and a length of 1 m was drawn through the µ‐PD method.^[^ ^132^ ^]^ Given that the color center defects in garnet crystals easily cause unexpected additional absorption, Sidletskiy et al. introduced a combinative strategy to enhance the light attenuation length of YAG fiber‐derived structured scintillator.^[^ ^133^ ^]^ By rational introduction of excess Al, optimization of thermal conditions of crystallization, and postgrowth annealing, fiber smoothness, and crystallization stability could be considerably improved, and a 380 mm light attenuation length could be achieved in a modified sample. YAP is also a promising scintillating scintillator for its high light yield and good mechanical and chemical properties. Via the µ‐PD method, Ce^3+^‐doped YAP fiber‐derived structured scintillator was grown by Alshourbagy et al.^[^ ^134^ ^]^ The diameters ranged from 0.5 to 2.5 mm, and the length can reach 150 mm. Under gamma‐ray excitation, corresponding photopeak locating at the 1191 ± 62 channels can be clearly observed in the spectrum of the 0.5% Ce^3+^‐doped sample.

### 4.3 Polymer Fiber‐Derived Structured Scintillators

Similar to glass, plastic scintillators show great feasibility for developing fiber‐derived structured scintillators. After years of development, various novel polymer fiber‐derived structured scintillators have been introduced. For example, Borshchev et al. demonstrated a promising fiber‐derived structured scintillator with a short decay time and a high light yield.^[^ ^135^ ^]^ Efficient activators and a special spectral shifter called nanostructured organosilicon luminophores were admixed to the styrene monomer and copolymerized as a cylindrical core rod. Then, the as‐made core–rod was dressed in two cylinders and drawn into multicladding fibers in a heating furnace. The characterization results indicated that structured scintillators obtain comparable light yields and much shorter decay times compared with reference commercially available fiber SCSF‐3HF and SCSF‐78. To improve further the scintillation performance of polymer fiber‐derived structured scintillator, which suffers from its intrinsic low density, *Z* number, and as a result, limited radiation stopping power, high *Z* particles have been selected and incorporated into polymer matrix with the strategy introduced in Section 2. In a recent work reported by Whittaker and co‐workers, three typical fiber‐derived structured scintillators embedded with different quantum dot particles were fabricated and investigated.^[^ ^136^ ^]^ Various types of quantum dots (CdS/Cd_0.5_Zn_0.5_S/ZnS, CdS/Cd_0.8_Zn_0.2_S/‐Cd_0.5_Zn_0.5_S/Cd_0.8_Zn_0.2_S/ZnS, and CdS) were copolymerized with a styrene monomer to synthesize fiber core preforms. Then, preforms were incorporated with PMMA tubes and drawn into fibers. Under 6 MV X‐ray radiation, all fibers showed a bright luminescence and a linear response in the range from 20 to 500 cGy.

**Figure 15:**
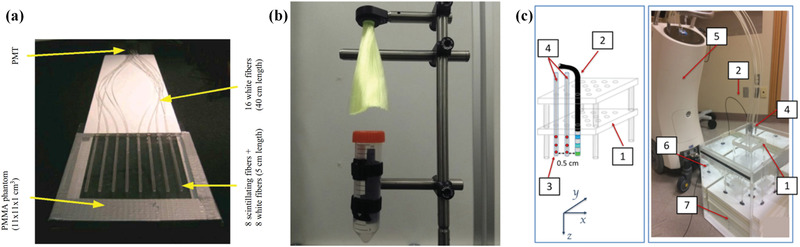
Constructed devices based on polymer fiber‐derived structured scintillators. a) Protype fiber dosimeter basing on BCF‐10 fiber‐derived structured scintillators for real‐time gamma‐ray and neutron monitoring on radiotherapy accelerators. b) The sensor device based on BCF‐20 structured scintillators toward alpha particle detection in liquids for application of radioisotope monitoring. c) Multipoint in vivo dosimeter based on BCF‐10, BCF‐12, and BCF‐60 fiber‐derived structured scintillators for application of high dose rate brachytherapy. a) Reproduced with permission.^[^ ^137^ ^]^ Copyright 2007, Elsevier. b) Reproduced with permission.^[^ ^139^ ^]^ Copyright 2019, Elsevier. c) Reproduced with permission.^[^ ^140^ ^]^ Copyright 2019, Wiley‐VCH.

Several polymer fiber‐derived structured scintillators have been studied in detail and even achieved commercialization, including BCF‐10, BCF‐12, BCF‐20, BCF‐60 (Saint‐Gobain, France) SCSF‐78, and SCSF‐81 (Kuraray, Japan). Benefiting from their advantages of fast response, flexibility, and low cost, many fiber‐based devices have been successfully constructed, targeting different applications (**Figure** **15**a‐c. For example, for real‐time gamma‐ray and neutron monitoring on radiotherapy accelerators, Bartesaghi et al. demonstrated a prototype fiber dosimeter basing on BCF‐10 fiber‐derived structured scintillator.^[^ ^137^ ^]^ Eight parallel BCF‐10 structured scintillators were embedded in a PMMA phantom and coupled to a photodetector. Under radiation from 6 MV photon and 6 MeV electron beams, the obtained response from the protype can fit the reference data with only a few percent of discrepancy. In another work performed by Leverington et al., a protype beam profile monitor was constructed with SCSF‐78MJ structured scintillators and tested at the Heidelberg Ion Therapy Centre.^[^ ^138^ ^]^ Double‐clad SCSF‐78MJ structured scintillators were wound into a multilayer ribbon, bonded between two PVC frames, and finally mounted to the 64 channels photodiode arrays. Its detector performance responding to four different ion therapy beams (protons, helium, carbon, and oxygen ions) were characterized, including signal‐to‐noise ratio, beam position resolution, beam with resolution, and signal amplitude resolution, showing great potential in practical application. Toward alpha particle detection in liquids for the application of radioisotope monitoring, Whittaker and co‐workers evaluated a range of commercially available and in‐house fabricated fiber‐derived structured scintillators.^[^ ^139^ ^]^ BCF‐20 structured scintillators were further selected and incorporated into sensor device, enabling alpha particle detection at solution activity as low as 0.42 Bq mL^‐1^. For application of high dose rate brachytherapy, Rosales and co‐workers demonstrated a multipoint in vivo dosimeter based on BCF‐10, BCF‐12, and BCF‐60 fiber‐derived structured scintillators.^[^ ^140^ ^]^ Three segments of different types of fiber‐derived structured scintillators were spliced by clear optical fibers and coupled to the photodetector to fabricate the protype dosimeter. In the phantom measurement using an ^192^Ir source, the difference between the test result and the standard data remained below 5% in the range of 0.5–6.5 cm. The signal‐to‐noise ratio remained above 5 for dose rates above 22 mGy s^‐1^. The results indicated that the device allows multipoint simultaneous dose measurement over a clinically useful distance range.

## 5 Conclusion and Overlook

Structured scintillators present exceptional performances compared with those of traditional scintillators, proving that “structure engineering” is a highly promising strategy. In this review, the state‐of‐the‐art development of this strategy for the development of new‐generation scintillators is presented. Based on their structure configuration, three kinds of typically structured scintillators, particle‐derived, array‐derived, and fiber‐derived structured scintillators, are introduced and reviewed.

In Section 2, particle‐derived structured scintillators are introduced and represent a new category of scintillator candidates. For particle–polymer structured scintillators, high *Z* number particles can be selected to increase the X‐ray or gamma‐ray stopping power, and as a result, the integrated radiation detection efficiency. In addition, by rationally combing the emitting polymer matrix and neutron‐sensitive particles, structured scintillators with gamma/neutron discrimination ability can be fabricated, indicating the potential of designing scintillators with unique functions by introducing various particles. Its ability to combine the properties of two or even more kinds of materials provides endless possibilities. For particle–glass structured scintillators, the participating crystalline particles could provide the active dopants with a periodic and low phonon energy environment, facilitating efficient radioluminescence. Though several encouraging particle‐derived structured scintillators have already been reported, the relatively weak scintillating performance remains the fundamental limitation of these candidates. Generally, a higher loading of the particles is preferred from the standpoint of scintillation performance, but it severely exacerbates the difficulty of material synthesis. To maintain transparency, the refractive index matching between the plastic/glass matrix and the participated particles is critical and challenging. Until now, to the best of our knowledge, only limited types of high loading structured scintillators have been reported, leaving much room for further research. For particle–polymer structured scintillators, quantum dots are of particular interest in terms of transparency and deposited energy harvest due to their unique size effect. Several problems, mainly aggregation, self‐absorption, and efficiency deterioration, currently need to be addressed while strategies such as in situ polymerization have been proven efficient in a few special cases, indicating possible solutions for future development. For particle–glass structured scintillators, increasing particle loading ratio, that is crystallinity, relies on carefully designing the glass matrix chemical composition and crystallization. Recently, several fully crystallized transparent glass ceramics have been reported, one of which exhibits outstanding scintillating performance. Broadening these types of special glass systems to the field of scintillators may be the mainstream of research.

In Section 3, the development of array‐derived structured scintillators is discussed. For the application of high‐resolution radioluminescence imaging, two popular strategies for fabricating self‐growth array‐derived scintillators are introduced: controllable film growth and unidirectional solidification of eutectic melt. The obtained structured scintillators show unique light‐guiding properties, enabling efficient suppression of light cross‐talk. Moreover, the utilization of a rationally designed template could further develop the controllable film growth strategy, facilitating a compact array structure and a high imaging resolution. After dozens of years of scientific research, CsI:Tl‐based array‐derived structured scintillators have been widely studied, and several commercial products are even available. Other materials, especially those of nanoarrays such as ZnO exhibit great potential but require more research. Beyond the existing ones, more material candidates are expected to expand the systems. Moreover, array‐derived structured scintillators with external coating are introduced. Via lithography and self‐assembly method, an array structure can be constructed on the surface of traditional scintillators, remarkably enhancing the light extraction efficiency between the scintillator and the photodetector. Large‐scale fabrication of these special structures with a low cost and a high repeatability presents the main hindrances. Although various techniques have been developed, none of them can fully satisfy all requirements. Among them, the nano imprint lithography and self‐assembly method acquire more attention while requiring further optimization. Toward ultrafast gamma‐ray detection, array‐derived structured scintillators composed of multiple high Z materials layers and fast photon emitters layers have been constructed. The combination of existing materials with specially designed structure provides an alternative method to push the time limit. Relatively, this emerging candidate is still in its infancy, with few works reported. More material choices along with novel structure design may open a rich field in the coming years.

In Section 4, the progress of the fiber‐derived structured scintillators is summarized. Benefiting from the advantage of plasticity, glass and polymer scintillator can be easily constructed into fiber configuration. Rare‐earth element‐doped fiber‐derived structured scintillators and multicomponent glass fiber‐derived structured scintillators as well as polymer fiber‐derived structured scintillators have aroused great interest and undergone rapid development. Furthermore, several polymer fiber‐derived structured scintillators are even commercially available and widely applied in device construction for practical applications. To meet the increasingly demanding requirements, which cannot be fulfilled with the existing products, plastic/glass fiber‐derived structure scintillators with a superior performance are desired. Combining the concept of particle‐derived structured scintillator with fiber‐derived structured scintillator presents the potential to overcome the bottleneck, with several exciting results recently reported. Remarkable progress could also be seen in the synthesis of crystal fiber‐derived structured scintillators. Various well‐known traditional crystal scintillators, including but not limited to BGO, YAG, LYSO, and YAP, have been successfully drawn into fiber shape. Nevertheless, crystal fiber‐derived structured scintillator development is seriously limited by fabrication. To date, very few high‐quality crystal fibers with a length of over a meter have been reported. Improving fiber quality, which currently focuses on modifying fiber drawing parameters such as drawing rates and crucible structure and manipulating the raw material ratio, especially those of volatile and doping, should be the research focus in the future.

From the overall point of view of the structured scintillator, several prospects are proposed: 1) The physical principles beyond the whole scintillation have not been fully revealed, limiting the theoretical studies and further material design. For traditional scintillators and structured scintillators, a more comprehensive understanding of the scintillation mechanism is urgently required. 2) Further focusing on structured scintillators, the relationship between the introduced structures and the performances has to be established, at least for several specific systems. To provide a guideline for structured scintillator design and synthesis, clearly understanding the influence of the introduced structures, from the basic structured units to the structure–scintillator interface then to the integrated structured scintillators, is indispensable. 3) Given that structured scintillators are emerging as a new type of material candidates, traditional fabrication methods face their intrinsic limits. Seeking novel manufacture technology may drive the development of new structured scintillators. 4) In addition to the structured scintillators introduced in this review, the “structure engineering” strategy enables the integrating other novel structures in the scintillators. Exploration of new types of structured scintillator candidates as well as combination of different types of structured scintillators are of great potential. 5) Structured scintillators have undergone a rapid development in the past decades, but the candidates that could be successfully translated to mature commercial products are still limited. From laboratory synthesis to industrial production, much needs to be done. In large‐scale production, quality and cost control are critical, requiring further optimization of the manufacturing technique.

## Conflict of Interest

The authors declare no conflict of interest.
